# C-type natriuretic peptide is a pivotal regulator of metabolic homeostasis

**DOI:** 10.1073/pnas.2116470119

**Published:** 2022-03-25

**Authors:** Cristina Perez-Ternero, Aisah A. Aubdool, Raj Makwana, Gareth J. Sanger, Roland H. Stimson, Li F. Chan, Amie J. Moyes, Adrian J. Hobbs

**Affiliations:** ^a^William Harvey Research Institute, Barts & The London School of Medicine & Dentistry, Queen Mary University of London, London EC1M 6BQ, United Kingdom;; ^b^Blizard Institute and the National Centre for Bowel Research, Barts & The London School of Medicine & Dentistry, Queen Mary University of London, London E1 2AT, United Kingdom;; ^c^University/British Heart Foundation Centre for Cardiovascular Science, The Queen’s Medical Research Institute, University of Edinburgh, Edinburgh EH16 4TJ, United Kingdom

**Keywords:** natriuretic peptide, G protein–coupled receptor, cardiometabolic disease, adipogenesis, thermogenesis

## Abstract

Thermogenesis and adipogenesis are tightly regulated mechanisms that maintain lipid homeostasis and energy balance; dysfunction of these critical processes underpins obesity and contributes to cardiometabolic disease. C-type natriuretic peptide (CNP) fulfills a multimodal protective role in the cardiovascular system governing local blood flow, angiogenesis, cardiac function, and immune cell reactivity. Herein, we investigated a parallel, preservative function for CNP in coordinating metabolic homeostasis. Global inducible CNP knockout mice exhibited reduced body weight, higher temperature, lower adiposity, and greater energy expenditure in vivo. This thermogenic phenotype was associated with increased expression of uncoupling protein-1 and preferential lipid utilization by mitochondria, a switch corroborated by a corresponding diminution of insulin secretion and glucose clearance. Complementary studies in isolated murine and human adipocytes revealed that CNP exerts these metabolic regulatory actions by inhibiting sympathetic thermogenic programming via G_i_-coupled natriuretic peptide receptor (NPR)-C and reducing peroxisome proliferator-activated receptor-γ coactivator-1α expression, while concomitantly driving adipogenesis via NPR-B/protein kinase-G. Finally, we identified an association between CNP/NPR-C expression and obesity in patient samples. These findings establish a pivotal physiological role for CNP as a metabolic switch to balance energy homeostasis. Pharmacological targeting of these receptors may offer therapeutic utility in the metabolic syndrome and related cardiovascular disorders.

## Significance

The global mortality, morbidity, and healthcare costs associated with cardiometabolic disease, including obesity, diabetes, hypertension, and dyslipidemia, are substantial and represent an expanding unmet medical need. Herein, we have identified a physiological role for C-type natriuretic peptide (CNP) in regulating key processes, including thermogenesis and adipogenesis, which combine to coordinate metabolic function and prevent the development of cardiometabolic disorders. This protective mechanism, which is in part mediated via an autocrine action of CNP on adipocytes, is underpinned by activation of cognate natriuretic peptide receptors (NPR)-B and NPR-C. This mechanism advances the fundamental understanding of energy homeostasis and glucose handling and offers the promise of improving the treatment of cardiometabolic disease.

The exponential increase in the prevalence of obesity and other drivers of the metabolic syndrome (e.g., diabetes, hypertension, dyslipidemia) represents a major unmet medical need (1, 2). Characterized by increased adipose tissue mass, inflammation, and insulin resistance, obesity is associated with a well-defined predisposition to cardiovascular disease (and cancer) (3). Sequestration of lipids by white adipose tissue (WAT) prevents detrimental ectopic accumulation, but in the longer term can trigger broader metabolic imbalance; in contrast, brown adipose tissue (BAT) promotes thermogenesis and improves the overall cardiometabolic phenotype (4). Thus, a better understanding of the molecular processes involved in lipid handling and energy balance should facilitate development of effective therapies for obesity, the metabolic syndrome and, more broadly, cardiovascular disease.

C-type natriuretic peptide (CNP) is a fundamental, paracrine mediator that coordinates cardiovascular homeostasis (5–11). Several disparate reports have hinted at a role for CNP in the regulation of metabolic function. Studies in isolated preadipocytes show that administration of CNP, or the analog CD-NP, stimulates lipid accumulation and expression of thermogenic markers via guanylyl cyclase-coupled natriuretic peptide receptor (NPR)-B (12, 13). Aligned to such cell-based studies, pharmacological administration of CD-NP in vivo worsens diet-induced obesity and increases body weight; yet, overexpression of the peptide in adipocytes or endothelial cells reduces adipocyte hypertrophy and improves glucose clearance without triggering lipolysis or altering body weight (14–17). Likewise, mice with an inactivating NPR-B mutation accumulate less WAT (18). Indeed, CNP and NPR-B have been linked to changes in feeding behavior, suggesting there might be a central component to any effects of the peptide on metabolism (19). In contrast, mice with genetic deletion of NPR-C, which functions in part to internalize and diminish natriuretic peptide bioactivity (20), have lower body weight and increased thermogenesis (21, 22), a phenotype thought to result from an increased endocrine influence of atrial natriuretic peptide (ANP) that stimulates thermogenesis via an NPR-A/PKG/p38 MAPK pathway. In the human adult population, a relationship between CNP and obesity has yet to be established and is complicated by effects on bone growth (23, 24). Nonetheless, epidemiological data in obese patients suggests a strong relationship between NPR-C expression and obesity/insulin resistance (25). However, large population studies suggest this is due to decreased secretion of ANP and brain natriuretic peptide (BNP) (26–28) rather than a causal role for NPR-C in reducing natriuretic peptide bioactivity (29).

Herein, we unite and explain these earlier, disconnected observations by demonstrating that CNP functions as a key regulator of energy homeostasis. Through the development of a unique transgenic mouse strain with inducible, global deletion of CNP, we reveal a pivotal multifaceted role for the peptide in governing adipogenesis, thermogenesis, and glucose utilization. Furthermore, we describe an association between CNP, NPR-C, and metabolic status in the adult human population. Such findings imply that targeting CNP signaling represents a means to pharmacologically manipulate energy expenditure, lipid metabolism, and glucose clearance in patients with cardiometabolic disease.

## Results

### Development and Characterization of a Global, Inducible CNP Knockout Mouse.

**Fig. 1.**
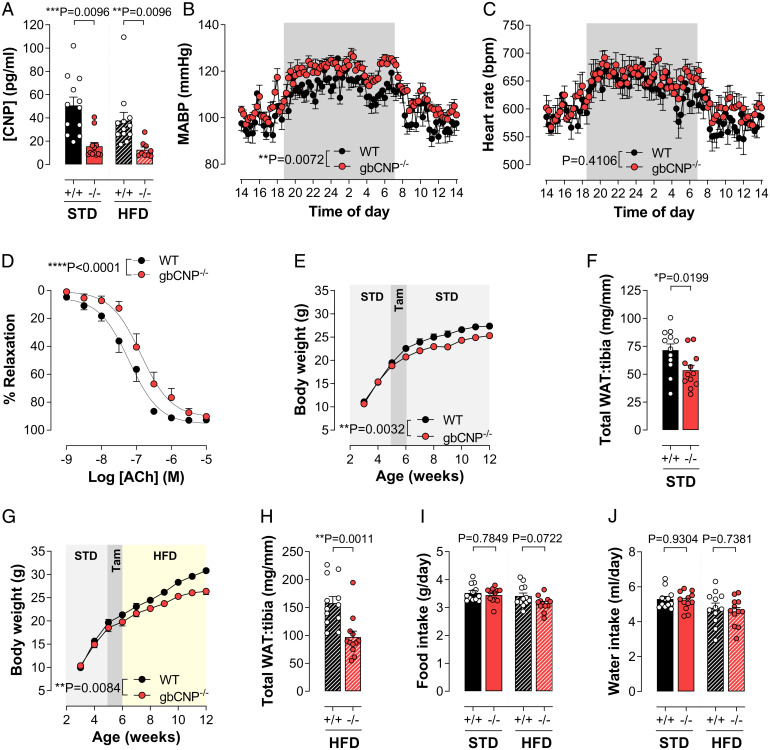
Body weight and WAT accumulation are markedly reduced in gbCNP^−/−^ mice in vivo. Plasma levels of CNP (*A*) in WT (^+/+^) and gbCNP^−/−^ (^−/−^) mice fed STD chow or HFD. Mean arterial blood pressure (MABP; *B*) and heart rate (HR; *C*) measured by radiotelemetry, and endothelium-dependent relaxation to acetylcholine in mesenteric arteries (ACh; *D*) in WT (^+/+^) and gbCNP^−/−^ (^−/−^) mice. Body weight and sum of all WAT (gonadal, perirenal, inguinal, and mesenteric) weight in WT and gbCNP^−/−^ animals fed STD chow (*E* and *F*) or HFD (*G* and *H*). Food (*I*) and water (*J*) intake. Data are represented as mean ± SEM *n* = 7 to 18. Statistical analysis by two-way ANOVA with Šídák post hoc test (*A*, *I*, and *J*), two-way repeated-measures ANOVA (*B*, *C*, *E*, and *G*), two-tailed Student’s *t* test (*F* and *H*). Each statistical comparison undertaken has an assigned *P* value (adjusted for multiplicity).

Administration of tamoxifen to CNP^flox/flox^ animals at an age of 5 wk resulted in global deletion of CNP (gbCNP^−/−^), compared to WT (i.e., gbCNP^+/+^) littermates, at the mRNA level from all tissues explored (6 wk after induction of gene deletion) (*SI Appendix*, Fig. S1*A*). Quantitative analyses of mRNA expression also revealed an efficient down-regulation of the gene expression (∼70%) in adipose tissue (*SI Appendix*, Fig. S1 *B–E*) and, while circulating CNP concentrations were significantly reduced by administration of tamoxifen (∼70%) (Fig. 1*A*), plasma levels of ANP and BNP were unaffected (*SI Appendix*, Fig. S1 *F* and *G*). Effective removal of the *Nppc* gene was confirmed at a functional level by an increase in mean arterial blood pressure without changes in heart rate variability (HRV) or electrocardiogram (ECG) in telemetered mice (Fig. 1 *B* and *C* and *SI Appendix*, Table S1) and by a reduction in endothelium-dependent vasorelaxation in isolated arteries (Fig. 1*D* and *SI Appendix*, Fig. S2), as reported previously in endothelium-specific CNP^−/−^ animals (11). Finally, gbCNP^−/−^ mice did not exhibit any significant change in organ weights compared to WT counterparts (*SI Appendix*, Fig. S3). In concert, these data confirm efficient, comprehensive removal of the *Nppc* gene in this new gbCNP^−/−^ mouse strain.

### Body Weight and WAT Accumulation Are Markedly Reduced in gbCNP^−/−^ Mice In Vivo.

**Fig. 2.**
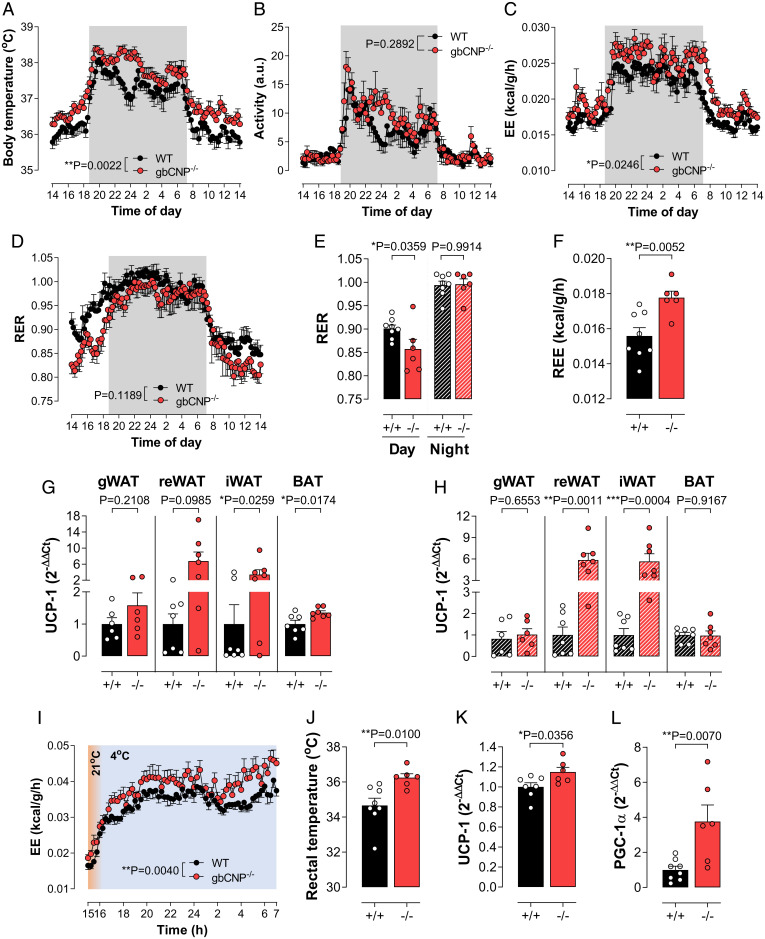
Body temperature and energy expenditure are overtly increased in gbCNP^−/−^ mice in vivo. Core body temperature (*A*), activity (*B*), EE (*C*), RER (*D* and *E*), and resting EE (REE; *F*) in WT (^+/+^) and gbCNP^−/−^ (^−/−^) mice. Expression of the thermogenic marker UCP-1 in the gWAT, reWAT, iWAT, and BAT in STD- (*G*) and HFD- (*H*) fed animals. EE (*I*) and rectal temperature (*J*) in WT (^+/+^) and gbCNP^−/−^ (^−/−^) mice following cold temperature (4 °C) challenge. Expression of the thermogenic markers UCP-1 and PGC-1α in BAT in gbCNP^−/−^ mice compared to WT (^+/+^) littermates (*K* and *L*). Data are represented as mean± SEM *n* = 6 to 8. Statistical analysis by two-way repeated-measures ANOVA (*A–D* and *I*), two-way ANOVA with Šídák post hoc test (*E*), or two-tailed Student’s *t* test (*F–H*, *J–L*). Each statistical comparison undertaken has an assigned *P* value (adjusted for multiplicity).

Following CNP deletion, gbCNP^−/−^ animals exhibited a significantly reduced weight gain over the following 6 wk and WAT accumulation compared to WT littermates, whether fed standard chow (STD) or a high fat diet (HFD) (Fig. 1 *E–H*); in a small number of animals, body weight was followed for 12 mo and confirmed the resistance to weight gain over the entire period in gbCNP^−/−^ mice (*SI Appendix*, Fig. S1*H*). This lower body mass, which was not a result of altered food or water consumption (Fig. 1 *I* and *J*) or activity (Fig. 2*B*), was matched by a striking reduction in the WAT, particularly apparent during high-fat feeding, in the gonadal (gWAT), peri-renal (reWAT), inguinal (iWAT), and mesenteric (mesWAT) regions without an alteration in BAT (*SI Appendix*, Fig. S1 *I–M*). Such data implied a major role for CNP in the regulation of energy homeostasis.

### Body Temperature and Energy Expenditure Are Overtly Increased in gbCNP^−/−^ Mice In Vivo.

Having identified that CNP governs body weight via functional effects on WAT deposition, further studies were conducted to explore the origins of these actions. In accord with the lower body weight and WAT mass, gbCNP^−/−^ animals had a significantly higher core body temperature (∼0.5 °C) in both the light and dark phase (Fig. 2*A*) that was not consequent to a greater activity (Fig. 2*B*) but rather an increased energy expenditure (EE; kcal utilized per gram body weight per hour) across a 24-h period (Fig. 2*C*). Moreover, measurement of gas exchange revealed a lower respiratory exchange ratio (RER) (Fig. 2 *D* and *E*), which was accounted for by an increase in O_2_ consumption in the absence of greater CO_2_ production (*SI Appendix*, Fig. S4 *A* and *B*). Indeed, in the dark (active) phase the RER in both WT and gbCNP^−/−^ animals was identical at ∼1.0, indicative of a predominantly carbohydrate-derived energy source. Notably, in the light (inactive) phase, while the RER in both strains was reduced, the gbCNP^−/−^ animals showed a markedly larger drop in RER, indicative of greater lipid utilization (Fig. 2*E*); this was mirrored by an increase in oxygen consumption that fits with lower glycolytic capacity in addition to increased thermogenesis (*SI Appendix*, Fig. S4*A*), resulting in a higher resting EE (Fig. 2*F*).

In light of the observations above, the expression of uncoupling protein (UCP)-1, a protein known to drive the beiging/browning of adipose tissue, was determined in the gWAT, reWAT, iWAT, and BAT. UCP-1 showed a significant increase in the browning sensitive adipose tissues (i.e., iWAT and BAT), but not in the lipid-storing adipose tissues (i.e., gWAT and reWAT) in gbCNP^−/−^ animals fed STD chow (Fig. 2*G*), and in the reWAT and iWAT in HFD-fed animals (Fig. 2*H*). These observations intimated that loss of CNP produces a prothermogenic phenotype characterized by increased body temperature, greater WAT metabolism, and sympathetic drive. To corroborate this premise, mice were exposed to a lower temperature (4 °C) to drive an intrinsic thermogenic response. This cold challenge induced a greater O_2_ consumption and energy expenditure in gbCNP^−/−^ animals (Fig. 2*I* and *SI Appendix*, Fig. S4 *C–E*), despite similar RER values that result from up-regulated use of carbohydrates as the predominant energy source following acute cold exposure (30). In this setting, the beiging of fat depots in gbCNP^−/−^ animals was significantly greater than WT counterparts (*SI Appendix*, Fig. S4 *F–J*), a phenotype accompanied by raised body temperature and increased expression of the thermogenic markers UCP-1 and PGC-1α in BAT (Fig. 2 *J–L*). Such data are consistent with the removal of a CNP-triggered brake on sympathetic drive that expedites a thermogenic reaction to cold.

### The Prothermogenic Phenotype in gbCNP^−/−^ Mice Is Recapitulated in Animals with Global NPR-C Deletion.

**Fig. 3.**
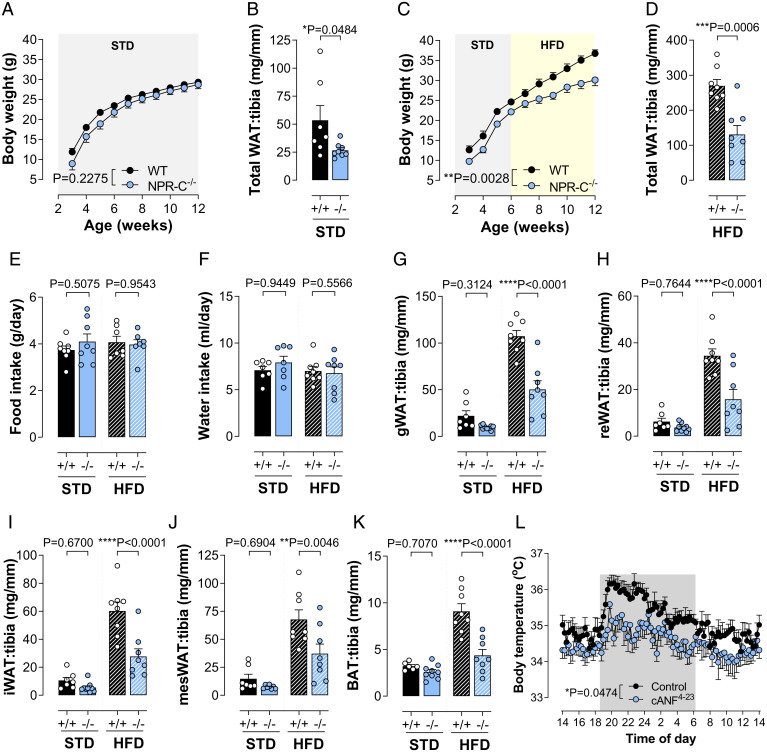
The prothermogenic phenotype in gbCNP^−/−^ mice is recapitulated in animals with global NPR-C deletion. Body weight and sum of all WAT (gonadal, perirenal, inguinal, and mesenteric) weight in WT and NPR-C^−/−^ animals fed STD chow (*A* and *B*) or HFD (*C* and *D*). Food (*E*) and water (*F*) intake, and gWAT (*G*), reWAT (*H*), iWAT (*I*), mesWAT (*J*), and BAT (*K*) in WT (^+/+^) and NPR-C^−/−^ (^−/−^) mice on STD chow or HFD. Body temperature and activity in WT animals following minipump infusion of cANF^4-23^ (0.4 mg/kg/d, subcutaneously) (*L*). Data are represented as mean ± SEM, *n* = 6 to 10. Statistical analysis by two-way repeated-measures ANOVA (*A*, *C*, and *L*), two-tailed Student’s *t* test (*B* and *D*), or two-way ANOVA with Šídák post hoc test (*E–K*). Each statistical comparison undertaken has an assigned *P* value (adjusted for multiplicity).

In order to establish which cognate receptor for CNP underpins the altered metabolic balance consequent to genetic ablation of the peptide, parallel studies were conducted in NPR-C^−/−^ mice. Remarkably, the augmented thermogenic profile observed following CNP deletion was mirrored precisely in animals lacking NPR-C, with reduced body weight and significantly lower WAT and BAT depots (Fig. 3); again, this was not a result of altered food intake (Fig. 3) nor increased plasma levels of natriuretic peptides as a consequence of reduced NPR-C–mediated clearance (ANP: WT, 272.7 ± 112.0 vs. NPR-C^−/−^, 270.2 ± 116.8 pg/mL, *n* = 3 to 5, *P* = 0.9890; BNP: WT, 126.9 ± 12.02 vs. NPR-C^−/−^ 114.9 ± 20.19 pg/mL, *n* = 5 to 6, *P* = 0.6078). To corroborate the contribution of NPR-C signaling to the regulation of energy metabolism, mice were infused with the selective NPR-C agonist cANF^4-23^ [atrial natriuretic factor(4-23); 0.4 mg/kg/d, subcutaneously] (31–36). Thus, selective activation of NPR-C resulted in a reduction in body temperature (∼0.6 °C) (Fig. 3*L*), confirming the importance of this cognate NPR in mediating the antithermogenic actions of CNP.

The prominent role for NPR-C, rather than NPR-B, in the metabolic regulatory role of CNP under conditions of thermal stress was further substantiated by assessment of receptor expression in WAT and BAT. Notably, expression of NPR-A and NPR-B remained constant in WAT and BAT regardless of diet or ambient temperature (with the exception of an up-regulation of NPR-B in the BAT at 4 °C) (*SI Appendix*, Table S2). In sharp contrast, expression of NPR-C was overtly changed in response to cold/heat exposure and fat content of the diet (*SI Appendix*, Table S2). These findings provide further evidence that fine control of NPR-C expression, and thereby signaling, is the primary mechanism underpinning the governance of thermogenesis by CNP.

### Glucose Clearance and Insulin Release Underlie the Effects of CNP on Lipid Handling.

In light of the greater energy expenditure and thermogenic response in vivo in gbCNP^−/−^ mice, we sought to determine whether this was explained by altered glucose clearance. Indeed, when maintained on STD chow, gbCNP^−/−^ animals displayed impaired glucose uptake (Fig. 5*A*); moreover, the glucose clearance in gbCNP^−/−^ animals mirrored that observed in WT mice with an HFD-induced impairment in glucose handling (*SI Appendix*, Fig. S5*B*). This appears to represent a maximal pathological change since gbCNP^−/−^ mice fed HFD did not deteriorate further with respect to glucose challenge (*SI Appendix*, Fig. S5*B*). The impaired glucose handling in gbCNP^−/−^ animals on STD chow was corroborated by increases in HbA1c (*SI Appendix*, Fig. S5*E*). However, this deficiency was not due to insulin insensitivity since the temporal dip in plasma (glucose) in WT and gbCNP^−/−^ animals administered bolus doses of insulin did not differ (*SI Appendix*, Fig. S5 *C* and *D*). Rather, the inability to regulate glucose levels efficiently was due to a reduced endogenous secretion of insulin, which was increased in response to the administration of a glucose bolus in WT but not gbCNP^−/−^ mice (*SI Appendix*, Fig. S5 *F*–*H*). These data reveal that the CNP-driven switch in favor of lipid utilization is underpinned by reduced insulin secretion and glucose uptake.

**Fig. 4.**
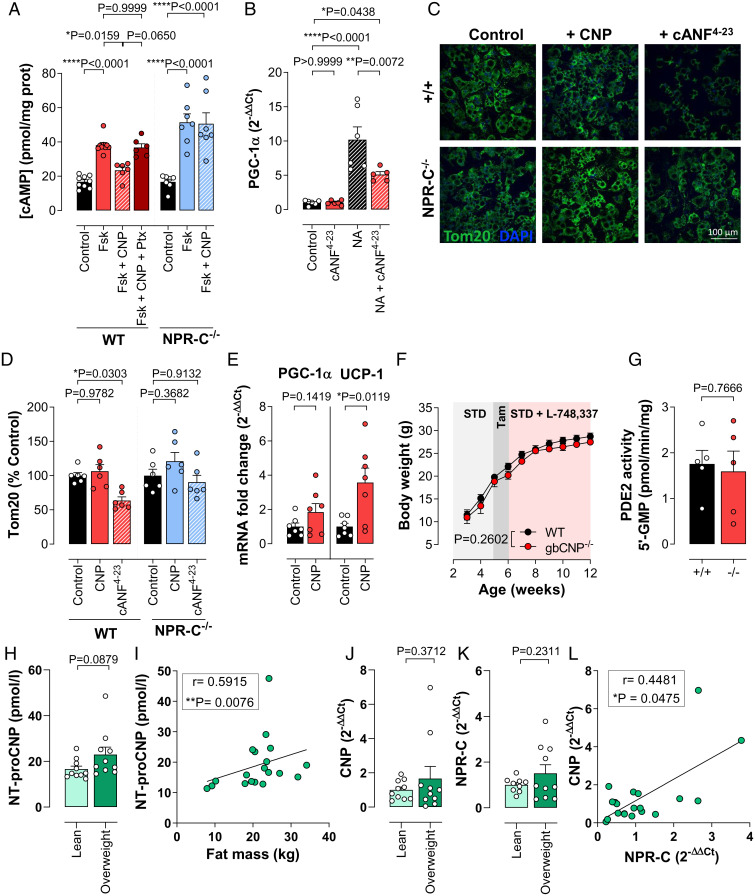
Inhibition of sympathetic thermogenic activity by G_i_-coupled NPR-C is responsible for the metabolic regulatory role of CNP. Forskolin (Fsk; 10 μM)-induced cAMP production in the absence and presence of CNP (100 nM) and *Pertussis toxin* (Ptx; 100 ng/mL) in primary adipocytes isolated from WT or NPR-C^−/−^ mice (*A*). PGC-1α mRNA expression in the presence and absence of NA (1 µM) and/or the selective NPR-C agonist, cANF^4-23^ (100 nM; *B*). Representative images (40x objective; *C*) and quantification (*D*) of Tom20 staining for mitochondria density in isolated adipocytes from WT and NPR-C^−/−^ mice in the absence and presence of CNP (100 nM) or cANF^4-23^ (100 nM). Results are shown as percentage of untreated control and expressed as the mean of >100 cells analyzed from three different fields per sample. Expression of the thermogenic markers PGC-1α and UCP-1 mRNA in the absence and presence of CNP (100 nM) in isolated adipocytes from WT mice (*E*). Effect of β_3_-adrenoreceptor blockage with L-748,337 (0.144 mg/kg/d) on body weight (*F*). PDE2 activity in brain homogenates (*G*). Plasma NT-proCNP concentrations (*H*). Correlation between the expression of plasma NT-proCNP and body fat mass in human patients (*I*). CNP (*J*) and NPR-C (*K*) mRNA expression from VAT from human patients. Correlation between CNP and NPR-C mRNA in VAT from human patients (*L*). Data are represented as mean± SEM. Statistical analysis by one-way ANOVA with Šídák post hoc test (*A–D*), two-tailed Student’s *t* test (*E*, *G*, *H*, *J*, and *K*), two-way ANOVA (*F*), or Spearman’s rank correlation (*I* and *L*). Each statistical comparison undertaken has an assigned *P* value (adjusted for multiplicity).

**Fig. 5.**
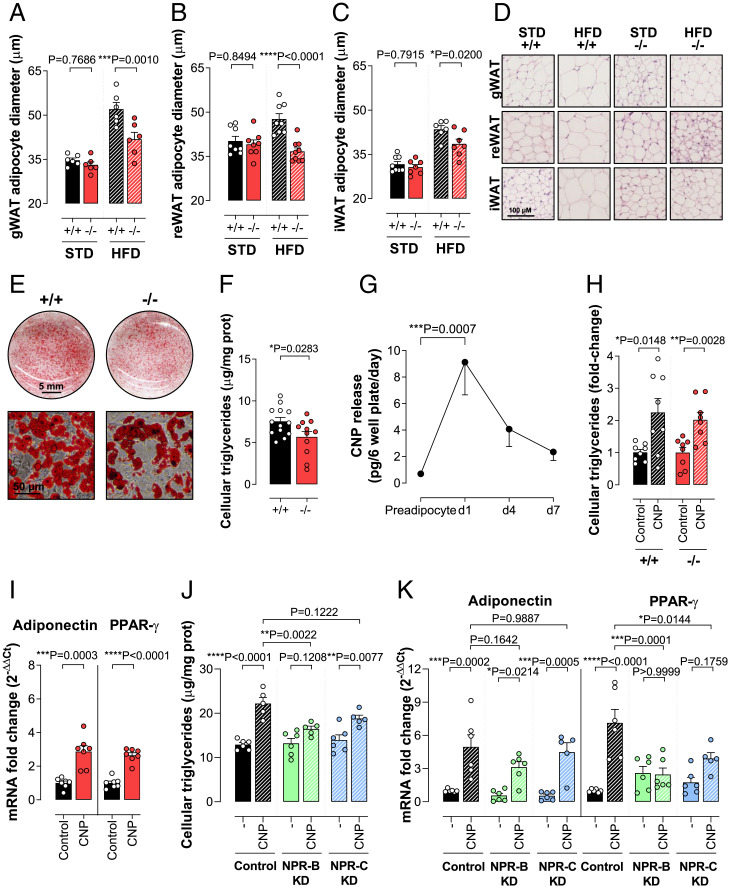
The prothermogenic actions of CNP deletion are associated with reduced adipogenesis. Mean adipocyte diameter in gWAT (*A*), reWAT (*B*), and iWAT (*C*) fat pads from WT (^+/+^) and gbCNP^−/−^ (^−/−^) mice fed STD chow or HFD. Representative images of H&E staining of WAT (*D*). Representative images of oil red-O staining from WT (^+/+^) and gbCNP^−/−^ (^−/−^) primary isolated adipocytes (*E*) and cellular triglyceride content (*F*). CNP in the culture media of murine primary adipocytes prior to the addition of the differentiation mixture (preadipocyte) and on day 1 (d1), day 4 (d4), and day 7 (d7) thereafter (*G*). Cellular triglyceride content in isolated murine adipocytes in the absence and presence of CNP (100 nM; *H*). Expression of the adipogenic markers adiponectin and PPAR-γ mRNA in isolated murine adipocytes in the absence and presence of CNP (100 nM; *I*). Cellular triglyceride content in human adipocytes following NPR-B (NPR-B KD) or NPR-C (NPR-C KD) knockdown in the absence (−) and presence of CNP (100 nM; *J*). Expression of the adipogenic markers adiponectin and PPAR-γ mRNA in isolated human adipocytes in the absence (−) and presence of CNP (100 nM; *K*). Data are represented as mean ± SEM, *n* = 5 to 13. Statistical analysis by two-way ANOVA with Šídák post hoc test (*A–C*) or two-tailed Student’s *t* test (*F–I*), or one-way ANOVA with Šídák post hoc test (*J* and *K*). Each statistical comparison undertaken has an assigned *P* value (adjusted for multiplicity).

In an attempt to further elucidate the mechanisms underpinning the impaired insulin release in gbCNP^−/−^ mice, we first examined plasma levels of somatostatin (SST), an inhibitory hormone that reduces insulin release from the pancreas. However, circulating SST concentrations did not change between WT and gbCNP^−/−^ animals, despite a subtle increase in HFD-fed animals (*SI Appendix*, Fig. S6*A*). Next, isolated pancreatic islets from WT mice were subjected to small-interfering RNA knockdown (siRNA) of NPR-B and NPR-C, and treated with CNP to evaluate insulin secretion in low (3.3 mmol/L) and high (16.7 mmol/L) glucose conditions. Successful silencing of the receptor expression was confirmed by qRT-PCR (*SI Appendix*, Fig. S6*B*). Despite an increase in the insulin release stimulated by high glucose, neither knocking down NPR-B/NPR-C nor stimulation with CNP had any effect on insulin secretion (*SI Appendix*, Fig. S6*C*). Subsequently, to rule out the possibility that the lack of effect of CNP was due to low background cAMP formation in an in vitro setting, pancreatic islets were stimulated with glucagon; again, CNP treatment and receptor knockdown showed no effect on insulin secretion (*SI Appendix*, Fig. S6*D*). Finally, we explored the possibility for NPR-B (i.e., cGMP)-mediated inhibition of phosphodiesterase (PDE)3 that might increase cAMP levels to promote insulin release in WT but not in gbCNP^−/−^ mice. Once more, we observed no effect in any of the experimental conditions (Fig. 2*D*). These data imply that there is an underlying mechanism that affects insulin secretion in vivo in gbCNP^−/−^ mice that could not be reproduced in vitro.

### Inhibition of Sympathetic Thermogenic Activity by G_i_-coupled NPR-C Is Responsible for the Metabolic Regulatory Role of CNP.

Having established a key role for CNP in regulating energy and glucose homeostasis, ex vivo cell-based studies were conducted to provide mechanistic insight into the underpinning mechanisms. In isolated, differentiated adipocytes (*SI Appendix*, Fig. S7) the response to “sympathetic activation,” through β-adrenoreceptor-coupled adenylyl cyclase stimulation with forskolin (assessed by cAMP generation) was attenuated by CNP in WT but not NPR-C^−/−^ cells (Fig. 4*A*); the importance of NPR-C G_i_-coupling was illustrated by the ability of *Pertussis toxin* to reverse these inhibitory actions of CNP in WT adipocytes (Fig. 4*A*). However, we did not observe a significant basal difference between WT and NPR-C^−/−^ adipocytes with respect to forskolin-induced cAMP accumulation, possibly due to low intrinsic NPR-C activity in adipocytes (Fig. 4*A*).

In addition, the selective NPR-C agonist cANF^4-23^ significantly reduced noradrenaline (NA)-induced up-regulation of the prothermogenic marker PGC-1α (Fig. 4*B*). Since PGC-1α is well-established to increase mitochondrial density (37), the effect of NPR-C deletion and activation was explored with respect to mitochondrial bulk. In isolated adipocytes, cANF^4-23^ produced a marked reduction in Tom20 staining, indicative of reduced mitochondrial density in WT cells but not in adipocytes from NPR-C^−/−^ mice (Fig. 4 *C* and *D*). Such a finding identifies an NPR-C–dependent action, via its G_i_-coupling (38), to inhibit local sympathetic (adrenergic) prothermogenic signaling (i.e., -β-adrenergic [β-AR]–triggered cAMP synthesis) that results in attenuated expression of PGC-1α and reduced mitochondrial density. Intriguingly, mitochondrial bulk did not increase despite the up-regulation of PGC-1α and UCP-1 expression in WT adipocytes in response to CNP treatment (Fig. 4*E*), possibly due to competing, concurrent activation of NPR-B (in addition to NPR-C). This phenomenon highlights a biological switch controlled by the environment. Under basal conditions, in which β-AR activation is minimal, the influence of NPR-C signaling is minimized, thereby revealing an NPR-B–dependent action of CNP that promotes PGC-1α (and UCP-1) activation. However, NPR-C–dependent inhibition of cAMP production predominates on a background of adrenergic stimulation. To confirm the contribution of a higher adrenergic drive to the adipose tissue of gbCNP^−/−^ mice, WT and gbCNP^−/−^ animals were treated with the specific β_3_-adrenoreceptor antagonist L-748,337 (39). As anticipated, β_3_-blockade nullified the body weight difference between WT and gbCNP^−/−^ mice (Fig. 4*F*).

While in vivo studies the with β_3_-adrenoreceptor antagonist L-748,337 and in isolated adipocytes confirm a local, autocrine action of CNP/NPR-C signaling on β-AR–triggered up-regulation of prothermogenic pathways, we also sought to provide evidence that this metabolic regulatory role of CNP might be underpinned by a central action on sympathetic outflow. To this end, we determined the PDE2 activity in brain homogenates to evaluate whether CNP deletion affected the NPR-B/cGMP/PDE2 pathway, which has been reported to play a central role in governing autonomic regulation of the heart (40, 41); however, PDE2A activity was not different between WT and gbCNP^−/−^ animals (Fig. 4*G*). Moreover, HRV, an index of autonomic balance with respect to cardiac function, was not modified in gbCNP^−/−^ mice compared to WT animals, nor following infusion of the selective NPR-C agonist cANF^4-23^ (0.4 mg/kg/d) (*SI Appendix*, Tables S1 and S3). These observations suggest that deletion of global CNP has little or no effect on central autonomic transmission (at least with respect to the indices measured herein) and that the metabolic actions of CNP are therefore likely exerted via a predominantly peripheral (i.e., local) effect at the cellular level (e.g., adipocyte).

Next, we sought to investigate the relevance of CNP in human obesity (patient anthropometric data can be found in *SI Appendix*, Table S4). Although there were no associations between circulating NT-proCNP (a more stable form of the cleaved prohormone) and the body mass index (BMI) (Fig. 5*G* and *SI Appendix*, Table S5), a positive correlation emerged between NT-proCNP and fat mass (Fig. 4*I* and *SI Appendix*, Table S5). In addition, although CNP and NPR-C mRNA expression was not influenced by the BMI in visceral fat samples (Fig. 4 *J* and *K*), a modest relationship between the expression of CNP and NPR-C in visceral adipose tissue (VAT) was identified (Fig. 4*L*). These data suggest a relationship between CNP expression and signaling pathways with the early stages of obesity in humans.

### The Prothermogenic Actions of CNP Deletion Are Associated with Reduced Adipogenesis.

Having validated the role of CNP in thermogenesis, we evaluated whether lower body weight of gbCNP^−/−^ mice was, in addition, the result of altered adipogenesis. Histological analyses of the gWAT, reWAT, and iWAT revealed that adipocytes from gbCNP^−/−^ animals were of significantly smaller diameter than those from WT littermates when fed an HFD to stimulate adipogenesis, whereas genotype did not significantly alter adipocyte size in animals on STD chow (Fig. 5 *A–D*); triglyceride content was also reduced in cultured adipocytes from gbCNP^−/−^ mice, mimicking the phenotype observed in vivo (Fig. 5 *E* and *F*). Next, we measured the CNP release from primary murine adipocyte cultures. CNP was detected in the culture media of preadipocytes at low levels, peaked on the first day after stimulation of differentiation, and slowly decreased thereafter (Fig. 5*G*). These data confirm that adipocytes produce and release CNP that acts in an autocrine fashion to promote adipogenesis; this concept was confirmed via exogenous addition of CNP, which was able to increase the lipid content of adipocytes from WT and gbCNP^−/−^ mice (Fig. 5*H*). In addition, CNP up-regulated the expression of the adipogenesis markers, such as the late transcription factor, peroxisome proliferator-activated receptor-γ (PPAR-γ), and the terminal differentiation marker, adiponectin, in WT adipocytes (Fig. 5*I*).

In order to assess whether a parallel regulatory system operates in humans, the effect of NPR-B or NPR-C knockdown was determined on the triglyceride content of isolated, differentiated human adipocytes. Successful receptor deletion was confirmed by a significant reduction in mRNA and protein expression (∼65% and ∼55% knockdown for NPR-B and NPR-C, respectively) (*SI Appendix*, Table S6). Paralleling observations in murine adipocytes, the addition of CNP to cultured human adipocytes increased adipocyte lipid loading and up-regulated expression of the adipogenic markers PPAR-γ and adiponectin in control adipocytes (Fig. 5 *J* and *K*). Following NPR-B knockdown, CNP-triggered triglyceride accumulation was reduced with a concomitant down-regulation of PPAR-γ expression; however, only CNP-mediated PPAR-γ expression was impaired as a consequence of NPR-C knockdown (Fig. 5 *J* and *K*). These findings intimate that while NPR-B signaling is fundamental for CNP-driven adipogenesis, NPR-C activation might be important at the early stages of adipogenesis.

### CNP/NPR-B/PKG Signaling Results in the Healthy Expansion of WAT In Vivo.

**Fig. 6.**
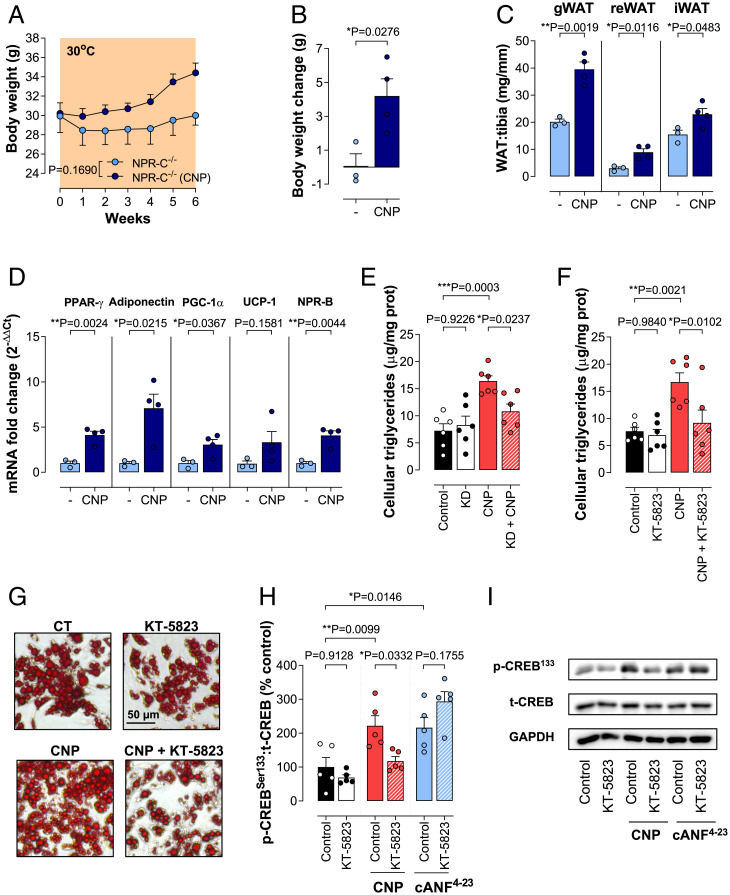
CNP/NPR-B/PKG signaling results in the healthy expansion of WAT in vivo. Body weight (*A*), body weight change (*B*), gWAT, reWAT, and iWAT (*C*), and mRNA expression of adipogenic (PPAR-γ and adiponectin), thermogenic (PGC-1α and UCP-1), and NPR-B (*D*) in iWAT of NPR-C^−/−^ mice in the absence and presence of CNP (0.2 mg/kg/d, subcutaneously). Effect of CNP (100 nM) on the triglyceride content of isolated murine adipocytes after NPR-B knockdown (KD; *E*) or in the absence and presence of the protein kinase G inhibitor KT-5823 (2 µM) (*F* and *G*). CREB phosphorylation in isolated murine adipocytes in the absence and presence of CNP, cANF^4-23^ (both 100 nM) and/or KT5823 (2 µM) (*H* and *I*). Data are represented as mean ± SEM, *n* = 3 to 6. Statistical analysis by two-way repeated-measures ANOVA (*A*) or one-way ANOVA with Šídák post hoc test (*E*–*H*), or two-tailed Student’s *t* test (*B*–*D*). Each statistical comparison undertaken has an assigned *P* value (adjusted for multiplicity).

To further explore which cognate receptor drives the CNP-mediated adipogenic response in vivo, NPR-C^−/−^ mice were housed at thermoneutrality (i.e., 30 °C) for 6 wk. In this setting, administration of CNP resulted in increased body weight gain and expansion of all WAT depots, revealing that the adipogenic response in vivo is governed by NPR-B signaling (Fig. 6 *A–C*). The study of mRNA expression of adipogenic (PPAR-γ and adiponectin) and thermogenic (PGC-1α and UCP-1) markers revealed an up-regulation of these processes in the iWAT (Fig. 6*D*). Interestingly, NPR-B expression was also up-regulated in this adipose tissue as a response to the increased circulating CNP. To confirm functional NPR-B dependency, murine adipocytes were stimulated with CNP after NPR-B knockdown (*SI Appendix*, Table S6) or in the presence or absence of the protein kinase (PK)G inhibitor KT-5823 (42). Indeed, the proadipogenic effect of CNP was completely abolished following NPR-B knockdown (Fig. 6*E*) and by the addition of KT-5823 (Fig. 6 *F* and *G*). Next, the downstream pathway involved in the adipogenic response activated by CNP was investigated. Both PKG and ERK signaling, downstream pathways linked to NPR-B and NPR-C, respectively, are known to trigger CREB by phosphorylation at Ser^133^, which is indispensable for the differentiation of preadipocytes to mature adipocytes (43–45). Furthermore, both CNP and cANF^4-23^ induced a significant CREB phosphorylation. Intriguingly, the phosphorylation induced by CNP, but not by cANF^4-23^, was completely inhibited by KT-5823, confirming that the adipogenic effect of CNP is conveyed predominantly via NPR-B/PKG activation, although some potential effects of NPR-C at the early stages of adipocyte differentiation may be important (Fig. 6 *H* and *I*), as observed in human adipocytes (see above).

## Discussion

CNP governs a number of cardiovascular processes, but a physiological role for CNP in the context of metabolic homeostasis has not been established; this is consequent to the well-defined role of the peptide in bone growth that causes dwarfism and early death in CNP-null mutant mice (46). Herein, we developed a unique transgenic mouse strain, with global inducible deletion of *Nppc* that circumvents these limiting issues and reveals a key role of the peptide in balancing energy metabolism by means of regulating thermogenesis, adipogenesis, and glucose clearance.

Characterization of this mouse model revealed an efficient gene deletion in all tissues and was functionally confirmed by increased blood pressure, as we and others have demonstrated previously in endothelium-specific CNP knockout animals (6, 11). Of note, the hypertensive phenotype resulting from global CNP deletion from 5 wk of age herein was observed in male mice, in contrast to the female-specific increase in blood pressure we observed following endothelium-specific *Nppc* gene deletion in utero (11), perhaps indicating males are able to functionally compensate by developmental up-regulation of NO signaling (47). Intriguingly, loss of CNP signaling resulted in an overt reduction in body weight gain and diminished accumulation of WAT; this was underpinned by a switch to thermogenic programming as indicated by a higher core body temperature, an abnormal preference for lipid utilization (over carbohydrate) for energy production, an up-regulation of UCP-1 and PGC-1α mRNA expression, and a clear visual beiging of WAT (and BAT) upon cold exposure. Importantly, although effective CNP deletion was confirmed in the brain, no clear changes in food intake were apparent (19, 48); likewise, central sympathovagal balance remained unchanged in gbCNP^−/−^ vs. WT animals (i.e., equivalent HRV, ECG, and PDE2 activity). These data intimate that the thermogenic effect of CNP deletion results predominantly from local (peripheral) mechanisms in the adipose tissue rather than as a consequence of altered central sympathetic drive; this conclusion was confirmed by the normalization of body weight gain in gbCNP^−/−^ in the face of β_3_-adrenoreceptor blockade.

In vitro data verified that the metabolic phenotypic changes produced by CNP deletion appear to be mediated primarily by an increased adrenergic action at the level of the adipocyte. CNP attenuated forskolin-induced cAMP production, an effect that was absent after *Pertussis toxin* treatment and in NPR-C^−/−^ adipocytes. Indeed, these antisympathetic actions of CNP pointed toward an NPR-C–, rather than NPR-B–, triggered pathway. Thus, it is straightforward to reconcile the effect of NPR-C activation mechanistically, because the receptor is known to contain G_i_ binding domains that inhibit adenylyl cyclase activity which, in turn, would be anticipated to offset adrenergic thermogenic programming and beiging of adipocytes. This concept was corroborated in vivo by a drop in core body temperature following infusion of the NPR-C–selective agonist cANF^4-23^. Such observations also established the direct signaling role for NPR-C in this context rather than as a result of its clearance activity (49); the latter function would be expected to cause an increase in body temperature by prolonging ANP/BNP bioactivity, thereby triggering the prothermogenic NPR-A/PKG/p38 MAPK pathway (21). In addition, NPR-C^−/−^ mice recapitulated the prothermogenic phenotype observed in gbCNP^−/−^ animals (principally following consumption of an HFD), indicating that CNP and NPR-C signal via a shared mechanism. The more modest metabolic phenotype in NPR-C^−/−^ animals fed STD chow is likely due to the smaller basal WAT depots as a result of a more pronounced, basal thermogenic profile resulting from constitutive NPR-C deletion (compared to the inducible CNP-null strain). In concert, these findings provide convincing evidence that CNP/NPR-C signaling plays a critical role as a brake to (sympathetic) thermogenic programming via direct inhibition of adenylyl cyclase. Indeed, this mechanism is emerging as a common instrument underpinning physiological roles of NPR-C in dampening sympathetic effects, including the regulation of blood pressure (50), heart rate (35), and cardiac function (41) (albeit the latter effect in part involves activation of PDE2).

Herein, we also demonstrate that CNP exhibits a pivotal control over adipogenesis, albeit primarily through NPR-B–dependent mechanisms (although blockade of NPR-C signal transduction also appears to contribute to the early stages of adipogenesis). In this setting, CNP triggers the downstream phosphorylation of CREB (at Ser^133^), which plays a critical role in the commitment of preadipocytes to differentiation (44, 51), a pathway that has been reported to be triggered by both NPR-C/G_i_ coupling (7) and cGMP/PKG signaling (52). Our data reveal that the NPR-B/PKG pathway is essential for CNP-induced adipogenesis, since pharmacological PKG blockade or NPR-B (but not NPR-C) knockdown in human and murine adipocytes in vitro completely blocks the adipogenic action of CNP. Furthermore, in vivo stimulation of adipogenesis (by CNP) was possible in NPR-C^−/−^ mice. In fact, infusion of CNP had a dramatic effect on the weight of NPR-C^−/−^ mice, up-regulating adipogenic and thermogenic markers in the subcutaneous adipose tissue, findings that fit with previous reports (13). These observations dovetail nicely with the transient up-regulation of CNP expression and release by adipocytes in the initial phases of adipogenesis. As such, we hypothesize that adipocytes are a key source of CNP, which drives differentiation in an autocrine manner without inhibiting PPAR-γ activity in the later stages of adipocyte maturation (53). Sequestration of lipids in newly generated adipocytes (hyperplasia) rather than by expansion of existing ones (hypertrophy) prevents a pernicious phenotype characterized by cellular hypoxia, necrosis, inflammation, and immune cell infiltration (54). Therefore, a role for CNP to facilitate the expansion of adipose tissue and increase in thermogenic markers—coupled with previous observations identifying important functions of CNP in promoting angiogenesis and (endothelial) proliferation while reducing (cardiomyocyte) hypertrophy and fibrosis and preventing inflammation (7, 8, 11)—intimate that CNP drives the healthy expansion of WAT, preventing the complications of metabolic syndrome and obesity.

Altered insulin signaling is also a well-stablished risk factor for cardio-metabolic disease (55). We observed a markedly impaired glucose clearance in global CNP-null mutants (the glucose insensitivity was masked in animals fed an HFD, presumably because CNP signaling is already compromised in these mice). This did not result from insulin insensitivity but rather reduced insulin release into the circulation following a glucose challenge. However, studies in isolated pancreatic islets suggest that it is not a direct effect of CNP on insulin secretion, either basally or stimulated by glucagon, nor is this phenomenon related to altered SST activity. Thus, further work is necessary to identify the underpinning mechanisms. Regardless, this influence of CNP on glucose clearance is likely to play an important role in the overall metabolic regulatory actions of the peptide.

Whether the established effects of other natriuretic peptides on metabolic homeostasis (21) also involve direct NPR-C–dependent signaling requires further elucidation. However, one reason underlying the reported thermogenic (i.e., opposing) actions of ANP/BNP might be related to distinctive cGMP compartmentalization in adipose tissue, as has been shown for the heart (56); alternatively, the ANP/BNP (via NPR-A) and CNP (via NPR-B/NPR-C) pathways may represent opposing mechanisms that permit fine-tuning of metabolic function. These possibilities too warrant more focused investigation, almost certainly involving the development and utilization of an adipocyte-restricted NPR-B–null mutant strain, which the present study lacks.

This metabolic regulatory role of CNP also has important implications for the pharmacological delivery of the peptide or mimetics. This is particularly true for heart failure, in which cachexia is a common and problematic symptom. Not only should interventions mimic the beneficial cardiac and vascular actions of CNP (i.e., promoting natriuresis, inhibiting the renin-angiotensin-aldosterone axis, and improving sympathovagal balance), but they should also promote adipogenesis in such a way to offset the severity of the cachexia without promoting detrimental ectopic (i.e., cardiac or vascular) deposition of lipid. In contrast, in other metabolic disorders, one might advocate blocking CNP to release a brake on sympathetic thermogenesis and reduce fat accumulation. However, this would likely be counterproductive in terms of cardiac and vascular health, increasing sympathetic drive and blocking the well-established beneficial actions of CNP in the heart and blood vessels.

Data from lean and overweight patients provide proof-of-concept as to the importance of the metabolic actions of CNP in the human population. While the size of this study was limited (a larger cohort of patients, including a wider range of BMI, would be needed to draw more definite conclusions), a clear relationship between CNP and NPR-C expression in the VAT is apparent, indicative of a fine signaling control of NPR-C in this context. Aligned to this finding, there is a positive correlation between the levels of NT-proCNP and the fat mass of patients, which fits with the proadipogenic capacity of CNP. However, we did not find an association between CNP and NT-proCNP with BMI. Whether such associations are more difficult to observe with respect to CNP, since it is predominantly an autocrine/paracrine mediator acting in a restricted, local manner (as opposed to the endocrine actions of siblings ANP and BNP), remains to be determined. Moreover, we did not observe an up-regulation of NPR-C expression in overweight patients, as has been previously reported; again, this may be due to an insufficient sample size or because patients with diabetes were excluded from our cohort (57).

**Fig. 7.**
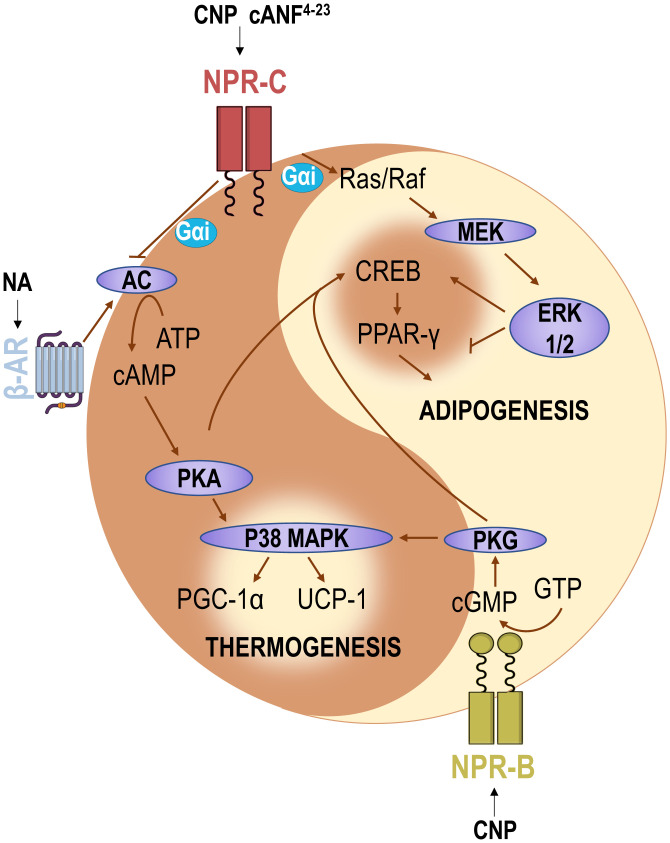
Schematic representation of the pathways involved in CNP control of energy homeostasis. Adenylyl cyclase, AC; adenosine triphosphate, ATP; cyclic adenosine monophosphate, cAMP; cyclic guanosine monophosphate, cGMP; cAMP-response element binding protein, CREB; extracellular signal-regulated kinase 1/2, ERK1/2; G_i_ protein α-subunit, G_αi_ guanosine triphosphate, GTP; mitogen-activated protein kinase kinase, MEK; p38 mitogen-activated protein kinases, P38 MAPK.

In sum, these data establish CNP as a fundamental regulator of lipid handling and energy homeostasis, coordinating adipogenesis and thermogenesis via actions at its two cognate receptors, NPR-B and NPR-C, triggering increases in cGMP and decreases in cAMP, respectively (Fig. 7). This metabolic regulatory role for CNP widens the critical cardio- and vasoprotective assignments of the peptide and heightens the therapeutic potential of drugs targeting CNP signaling in cardiovascular disease, including those associated with metabolic dysfunction.

## Materials and Methods

### Ethical Permission.

All animal studies conformed to the UK Animals (Scientific Procedures) Act of 1986 and had approval from a local (School of Medicine and Dentistry) Animal Welfare and Ethical Review Body. Experiments on human samples were approved by the London–City Road & Hampstead Research Ethics Committee (REC 15/LO/2127) and the Lothian National Health Service Research Scotland Human Annotated BioResource (15/ES/0094); all samples were collected under written informed patients’ consent.

### Generation of an Inducible, gbCNP^−/−^ Mouse.

*Nppc* LoxP-flanked mice (CNP^flox/flox^) were developed in house as described elsewhere (11) and crossed with a tamoxifen-inducible universal Cre-deleter line (B6.Cg-Tg[UBC-cre/ERT2]1Ejb/1J; The Jackson Laboratory). CNP gene deletion was induced by tamoxifen (40 mg/kg, intraperitoneally) injection of 5-wk-old CNP^flox/flox.UBC-cre/ERT2^ male animals (i.e., gbCNP^−/−^) for 5 consecutive days.

In some experiments, constitutive, global NPR-C knockout male mice (NPR-C^−/−^; kind gift of O. Smithies, University of North Carolina, Chapel Hill, NC; C57BLK6 background) were utilized to understand receptor specificity of CNP signaling. WT littermates (i.e., gbCNP^+/+^.Cre^+^ administered tamoxifen or NPR-C^+/+^) were used throughout.

### Metabolic Studies.

Mice were singly housed in a PhenoMaster metabolic cage system (TSE Systems) for 5 consecutive days with ad libitum access to food and water. VO_2_, VCO_2_, RER, spontaneous locomotor activity, and food and water intake were measured. Body temperature was recorded in conscious unrestrained mice using radiotelemetric transmitters (TA-F10; Data Sciences International). Further information can be found in *SI Appendix*.

### Glucose Metabolism.

For the analysis of glucose tolerance and insulin sensitivity, 12-wk-old mice were fasted for 6 or 3 h, respectively. Glucose (2 g/kg) or insulin (0.5 UI/kg; Novo Nordisk) were administered intraperitoneally, and blood glucose concentrations measured via the tail vein with a Roche Accu-Chek Aviva glucose monitor system (Roche).

### Protein and mRNA Quantification and Immunohistochemistry.

These were conducted according to standard protocols. Further information can be found in *SI Appendix*. Primer sequences used for RT-qPCR analysis can be found in *SI Appendix*, Table S7.

### Adipocyte Culture.

Primary adipocyte cultures were obtained from the stromal vascular fraction of mouse inguinal fat pads or human VAT. Specific protocols for the different cell treatments are detailed in *SI Appendix*.

### Quantification and Statistical Analysis.

All data are reported as mean ± SEM. Statistical analyses were conducted using GraphPad Prism (v8; GraphPad Software). Normal distribution of the data was confirmed using a Shapiro–Wilk test. For comparison of two groups of data, a two-tailed, unpaired Student’s *t* test was used. When comparing three or more groups of data, one-way or two-way ANOVA followed by a Šídák multiple-comparisons test was used with adjustment for multiplicity. *P* < 0.05 was considered statistically significant and the *P* values presented in each figure indicate all comparisons undertaken.

## Supplementary Material

Supplementary File

## Data Availability

All study data are included in the main text and *SI Appendix*.
