# Supplementary material for: C-type natriuretic peptide is a pivotal regulator of metabolic homeostasis
Source: Proc Natl Acad Sci U S A. 2022 Mar 25;119(13):e2116470119. doi: 10.1073/pnas.2116470119 (PMC9060477; doi:10.1073/pnas.2116470119)
Supplement: Supplementary File [file pnas.2116470119.sapp.pdf]

SUPPLEMENTAL MATERIAL

EXPANDED MATERIALS & METHODS

Genotyping and husbandry

The genotype of the animals was confirmed by PCR of genomic DNA extracted from ear biopsies (wild type band 842 bp; knockout band 956 bp). Successful gene deletion in the inducible colony (i.e. gbCNP$^{-/-}$) was confirmed by PCR by a reduction in CNP mRNA in all major tissues over a period of 6 weeks (***Figure S1A***).

Mice were group-housed in a controlled environment (20.37±0.11°C, 47.08±0.74% relative humidity), with a 12:12-h (7:00 a.m.-7:00 p.m.) light-dark cycle and free access to food and water. To mimic human dietary patterns driving obesity, mice were switched to a high fat diet at 6 weeks of age (HFD, 40% from kcal; TD.88137, Envigo, Indianapolis, USA) or continued to receive standard chow (STD; 5058, LabDiet, St. Louis, USA). Food intake and body weight were monitored weekly. Adipose tissue and other relevant organs were collected at 12 weeks of age, their weight normalized to tibia length (***Figure S3***), and snap frozen for further analyses.

NPR-C$^{-/-}$ mice were the kind gift of Prof O. Smithies (University of North Carolina at Chapel Hill, Chapel Hill, NC). Only male animals were used in this study.

Human samples

Human blood samples and visceral adipose tissue samples were collected from subjects undergoing elective abdominal surgery at the Royal Infirmary of Edinburgh, The Queen's Medical Research Institute, University of Edinburgh. Male (15%) and female (85%) donors aging 54.9 ± 2.6 years were included in the study. Sex-matched number for participants were included in each body mass index group. Patient anthropometric information

is summarized in ***Table S4***. Participants with diabetes or thyroid disease, or undertaking anti-diabetic treatment were excluded from the study.

Visceral adipose tissue samples for *in vitro* studies were obtained from stomach biopsies of patients undergoing bariatric surgery at Homerton University Hospital, London.

Metabolic studies

Metabolic studies were conducted in a Phenomaster metabolic cage system (TSE Systems, Thuringia, Germany). Respiratory exchange ratio (RER) and energy expenditure (EE) were calculated using the following equations: RER = VCO_2/VO_2, EE (kcal/h) = (3.815 + 1.232*RER)*VO_2. All calorimetric data presented were normalized to the body mass.

To evaluate the adaptive thermogenic response of mice below or at thermoneutrality, WT and gbCNP$^{-/-}$ animals were housed at 4°C for 16 hours. Rectal temperature was measured at the end of the cold challenge, after acquiring calorimetric parameters, and adipose tissue was collected for mRNA analyses (as below). In other studies, NPR-C$^{-/-}$ mice were implanted with osmotic mini-pumps (1004 and 1002; Alzet, Cupertino, CA) delivering CNP (0.2 mg/kg/d; s.c.) and were maintained at 30°C for 6 weeks. Alternatively, wild type mice were implanted with osmotic mini-pumps (1007D; Alzet, Cupertino, CA) delivering cANF$^{4\text{-}23}$ (0.4 mg/kg/d; s.c.) to assess changes in body temperature by radiotelemetry (as below) in singly housed animals at 21°C over a period of 7 days. Finally, to assess the effect of adrenergic drive on body weight, mice were implanted with osmotic mini-pumps (1004 and 1002; Alzet, Cupertino, CA) delivering the β_3-adrenoreceptor antagonist L-748,337 at a dose known to inhibit β_3-adrenoreceptor *in vivo* (0.144mg/kg/d; s.c.) (1).

Adipose tissue browning densitometric analyses

Browning of adipose tissue was assessed by densitometry using Image Fiji software (Image J; NIH, USA). Both contralateral fat pads for every adipose tissue depot were

measured and mean optical density was corrected for the background to normalize for differences in illumination.

Radiotelemetry analyses

Blood pressure, heart rate, electrocardiogram (ECG) and body temperature were recorded in conscious unrestrained mice using radiotelemetric transmitters (HD-X11, TA11 PA-C10 and TA-F10; Data Sciences International, St. Paul, Minnesota, USA). The transmitter blood pressure/heart rate catheter was implanted through the carotid artery and temperature sensors in the abdominal cavity of 5-week old mice. Blood pressure, heart rate, ECG and core body temperature were sampled from singly-housed 12-week old animals for 2 min every 15 min for a minimum of 60 hours. Data is expressed as an average for each time point for each mouse over 2 consecutive days of recording. Heart rate variability (HRV) and ECG parameters were analyzed as previously described (2) from six waveforms (2 min length) selected from periods of inactivity, using the HRV and ECG modules of Chart 8.1 (ADInstruments).

Vascular reactivity

Vascular reactivity was assessed in mesenteric arterial rings obtained from 12-week-old wild-type and gbCNP$^{-/-}$ mice using tissue bath pharmacology, as we have previously described (2).

Central PDE2 activity

Central nervous system phosphodiesterase (PDE) 2 activity was assessed in whole brain homogenates by measuring the ANP (1 μM)-induced production of 5′-GMP in the absence and presence of the selective PDE2 inhibitor BAY 60-7550 (1 μM) using a commercially available ELISA (Direct cGMP; Enzo Life Sciences, Farmingdale, USA).

Plasma biochemical analyses

Blood was obtained from anesthetized 12-week old mice by cardiac puncture and centrifuged at 18620 g for 2 min at 4°C. Plasma was used for the determination of glycated hemoglobin (HbA1c; Cloud-Clone, Houston, USA), BNP (Phoenix Pharmaceuticals, Karlsruhe, Germany) and somatostatin (SST; Cloud-Clone, Houston, USA). ANP and CNP plasma concentrations were determined after extraction with C18 columns using commercially available ELISA (Phoenix Pharmaceuticals, Karlsruhe, Germany). Insulin was measured with a commercially available kit (Merk-Millipore, UK) in plasma collected after 6 h fasting and 30 min after the administration of 2 g/kg glucose (i.p.). EDTA plasma from patients was used for the assessment of NT-proCNP levels with an ELISA kit (Biomedica, Vienna, Austria).

mRNA expression

Total mRNA was extracted using a RNeasy Lipid Tissue Mini Kit from adipose tissue or RNeasy Mini Kit from cells in culture (Qiagen, UK), according to the manufacturer's instructions. mRNA quality and quantity were assessed by absorption ratios of 260/280 nm and 260/230 nm using a NanoDrop spectrophotometer (Thermo Scientific, MA, USA). 1 µg of mRNA was then converted to cDNA by reverse transcription (Applied Biosystems, Life technologies Ltd, UK). 10 ng of cDNA was used for qRT-PCR analyses using PowerUp™ SYBR™ Green (Applied Biosystems, Life technologies Ltd, UK) and gene-specific primers listed in the ***Table S7*** on a Bio-Rad CFX96 Connect Real-Time PCR detection system (Bio-Rad, California, USA). Relative expression is represented as fold change of the wild type (tissue samples) or control (cell culture) using the cycle threshold (Ct) expressed as $2^{-\Delta\Delta Ct}$ relative to house-keeping gene *Rpl-19* levels. All qRT-PCR reactions were performed in duplicate.

Histology

Adipose tissue was fixed overnight in 4% paraformaldehyde at 4°C, dehydrated, embedded in paraffin, and sliced into 4 µm-thick sections. Hematoxylin-eosin (H&E) staining was performed to evaluate the adipocyte size. Images were obtained using a ×40 objective

by microscopy (NanoZoomer S210 Slide Scanner; Hamamatsu, Japan), and adipocyte size was measured using Fiji software (Image J; NIH, USA) equipped with the Adiposoft plugin. All adipocytes per section were measured and adipocyte diameter was represented as an average of all animals studied.

Primary cell isolation and cultures

Mouse primary adipocyte cultures were obtained from the stromal vascular fraction of the inguinal fat pads. 12-week old wild type and transgenic mice (i.e. gbCNP$^{-/-}$ and NPR-C$^{-/-}$) were used for this purpose. Adipose tissue was minced and digested for 30 min at 37°C with type 1 collagenase (1 mg/ml; Invitrogen, UK) in Hank's Balanced Salt Solution containing 7.5% bovine serum albumin (BSA fraction V; Fisher BioReagents, UK). The digested tissue was filtered through a nylon mesh (70 µm) and the pellet washed thrice. Pre-adipocytes thereby obtained were maintained in DMEM/F-12 media supplemented with 10% foetal bovine serum, 170 nM insulin and 150 µM sodium ascorbate in an incubator with 5% CO_2 humidified atmosphere at 37°C. Adipocytes from human visceral adipose tissue were obtained and maintained as described above, but the media was supplemented with 17 µM pantothenate and 33 µM biotin. After 7 days, differentiation was induced by adding an adipogenic cocktail containing 0.5 mM 3-isobutyl-1-methylxanthine (IBMX), 250 nM dexamethasone, 125 µM indomethacin and 1 nM thioredoxin in DMEM/F-12 growth media (3). 48 h thereafter, IBMX was removed from the media and cells were allowed to differentiate into mature adipocytes. Successful differentiation was confirmed qualitatively by observing lipid droplet formation using a light microscope and quantitatively by measuring mRNA levels of adipogenic markers (PPAR-γ, adiponectin and Pref-1) prior to and after differentiation at day 7 (***Figure S6***). All experiments were performed after 7 days of differentiation unless stated otherwise. CNP in the culture media was determined after extraction with C18 columns using a commercially available ELISA (Phoenix Pharmaceuticals).

cAMP measurement

The ability of CNP (100 nM) to inhibit forskolin (Fsk, 10 µM)-induced cyclic adenosine monophosphate (cAMP) production was evaluated in fully differentiated adipocytes obtained from WT and NPR-$C^{-/-}$ mice. 16 h pre-incubation with pertussis toxin (Ptx, 100 ng/ml) was used to investigate NPR-C/G_i coupling. After 20 min, cells were lysed with 1% triton-X/1M HCl, and the cAMP concentration measured by ELISA (Enzo Life Sciences, Farmingdale, USA). cAMP levels were normalized to the protein concentration in the lysate (BCA, Thermo Scientific, MA, USA).

***In vitro* study of NPR-C mediated inhibition of thermogenesis**

To investigate the effects of NPR-C/G_i inhibition on the PKA-mediated thermogenesis, murine adipocytes were incubated with 100 nM $cANF^{4-23}$ in the presence or absence of 1 µM noradrenalin in serum-free media for 4 h. mRNA was then isolated and used for qRT-PCR analyses (as above).

***In vitro* study of adipogenesis and mitochondrial mass**

CNP (100 nM) was added daily to the differentiation cocktail in WT, $gbCNP^{-/-}$ and NPR-$C^{-/-}$ derived adipocytes. In some experiments, the PKG inhibitor KT-5823 (2 µM) was added concurrently with CNP. After 7 days, cells were lysed with 5% Nonidet P-40/water and triglyceride content measured by a colorimetric kit (Abcam, UK). Triglyceride content was normalized by the protein concentration in the sample assessed by BCA (BCA, Thermo Scientific, MA, USA). Alternatively, 4% paraformaldehyde-fixed adipocytes were stained with oil red-O and examined visually. To determine mitochondrial mass, 4% paraformaldehyde-fixed adipocytes were permeabilized with 0.2% Triton X-100 for 5 min, blocked with 10% normal goat serum and 1% bovine serum albumin (BSA) for 45 min, and incubated with anti-Tom-20 antibody (1:200; Santa Cruz Biotechnology, CA, USA) for 2 h at room temperature, followed by the appropriate secondary antibody. Nuclei were stained with DAPI. Sections were

visualized using a Zeiss LSM 880 with FAST Airyscan (Zeiss, Germany) under a 40X objective.

In vitro study of CNP regulation of metabolic markers

To investigate the effect of CNP on the expression of different markers of adipogenesis and thermogenesis, 100 nM CNP was added daily to the media during the 7 days of the differentiation phase. mRNA was then isolated and used for qRT-PCR analyses (as above).

In vitro silencing of NPR-B and NPR-C expression

Human and mouse preadipocytes were grown to 70% confluence and then transfected with siRNA (10 nM) targeting NPR-B or NPR-C using lipofectamine 2000 (1 µg/ml) for 4 h. A non-sensical RNA sequence (10 nM) was used in the control wells. Then, cells were washed and an adipogenic cocktail (as above) was added. In some wells, CNP (100 nM) was added daily to the differentiation cocktail.

In vitro treatments for CREB phosphorylation

Adipocytes isolated from the inguinal fat pad and differentiated for 7 days were used to investigate CNP signaling in adipose tissue. Cells were incubated with DMEM/F12 for 4 h and then stimulated with 100 nM CNP or $cANF^{4-23}$ for 30 min. Some cells were incubated with the PKG inhibitor KT-5823 (2 µM) during the last 30 min prior to stimulation. Cell lysates were used to evaluate protein expression by Western blot.

Immunoblotting

Primary cell cultures were homogenized in ice-cold sodium dodecyl sulfate (SDS) lysis buffer (50 mM tris base, 10% glycerol, 2% SDS) containing a protease and phosphatase inhibition cocktail (Invitrogen, UK). Lysates were centrifuged (18620 g, 20 min, 4°C) and supernatants were used to determine protein concentrations (BCA; Thermo Scientific, MA, USA). 20 µg of protein lysate were separated in 10% SDS-PAGE and transferred onto

polyvinylidene fluoride membranes. The membranes were incubated overnight with the antibodies: anti-Phospho-CREB (Ser133) (1:1000; Cell Signaling Technology, MA, USA), anti-CREB (1:1000; Cell Signaling Technology, MA, USA) or GAPDH (1:4000; ThermoFisher Scientific, UK). Then membranes were incubated with secondary antibodies and developed by chemiluminescence (Millipore, UK) in a chemiluminescence imaging system (GeneGnome XRQ; Syngene, UK). Densitometry analyses were performed with Image Fiji software (Image J; NIH, USA).

Pancreatic islet isolation and treatments

Pancreatic islets were isolated from wild-type mice as previously described (4). Viable pancreatic islets were subjected to *in vitro* NPR-B or NPR-C RNA silencing as explained above. Islets were left to recover overnight and equilibrated in low glucose buffer (3.3 mmol/l) for an hour. Then, they were changed to high glucose buffer (16.7 mmol/l) alone or containing CNP (100 nM). In some cases, glucagon (10 nM) was added to the low glucose buffer to increase background cAMP formation, alone or in the presence of CNP (100 nM) and/or the PDE3 inhibitor milrinone (10 µM). Samples were extracted as previously described (4) and used to measure insulin release by ELISA (Merk-Millipore, UK).

SUPPLEMENTAL FIGURES

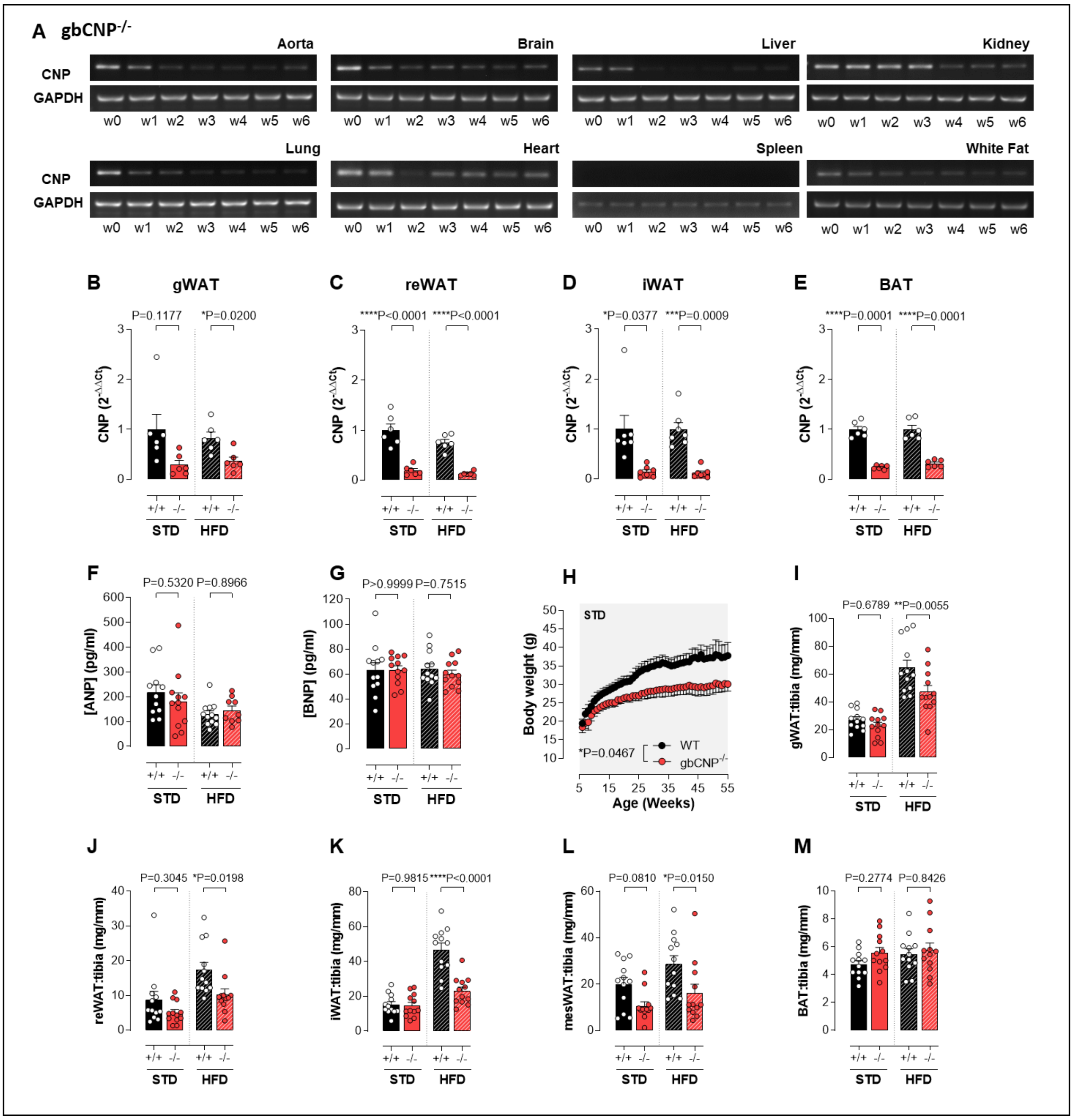

Figure S1: Characterization of a global, inducible CNP knockout mouse

PCR-amplified products from mRNA for CNP and GAPDH showing stable global deletion of CNP mRNA up to 6 weeks (w6) in global CNP knockout after tamoxifen-induced gene deletion (gbCNP$^{-/-}$; **A**) compared to the expression prior to tamoxifen administration (w0). CNP mRNA expression in gonadal (gWAT; **B**), peri-renal (reWAT; **C**) and inguinal (iWAT; **D**) white adipose tissue and brown adipose tissue (BAT; **E**) in wild type (+/+) and global CNP knockout (gbCNP$^{-/-}$; -/-) mice on standard chow (STD) or high fat diet (HFD) 6 weeks after gene deletion induction. Plasma levels of atrial natriuretic peptide (ANP; **F**), brain natriuretic peptide (BNP;

G) from chow (STD) and high fat diet (HFD) fed animals. Body weight in WT and gbCNP$^{-/-}$ mice over 12 months on standard chow (**H**). Gonadal (gWAT; **I**), peri-renal (reWAT; **J**), inguinal (iWAT; **K**) and mesenteric (mesWAT; **L**) white adipose tissue and brown adipose tissue (BAT; **M**) in wild type (WT, +/+) and global CNP knockout (gbCNP$^{-/-}$, -/-) mice on standard chow (STD) or high fat diet (HFD). Data are represented as mean±SEM. *n*=5-12. Statistical analysis by two-way analysis of variance with Šídák post hoc test. Each statistical comparison undertaken has an assigned *P* value (adjusted for multiplicity).

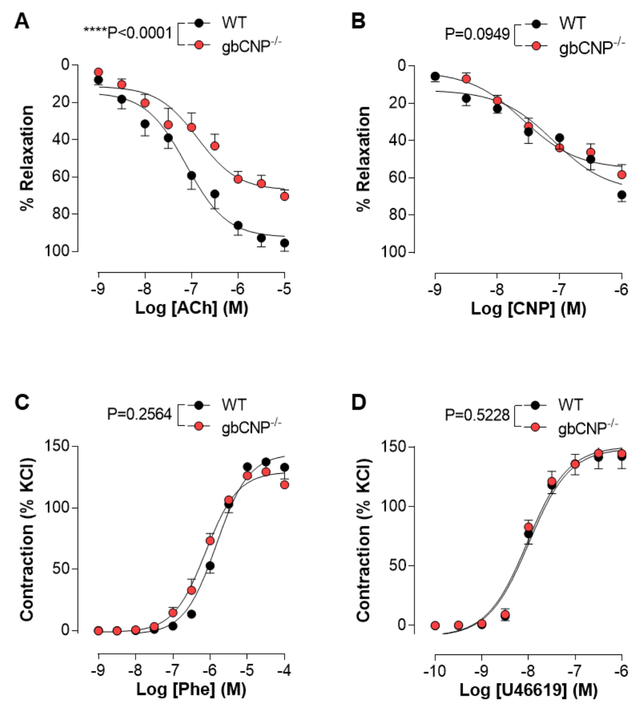

Figure S2: Global deletion of CNP diminishes endothelium-dependent vasorelaxation in mesenteric arteries

Endothelium-dependent relaxation to acetylcholine (ACh) was significantly reduced in mesenteric arteries from global C-type natriuretic peptide knockout ($gbCNP^{-/-}$) mice compared to wild type (WT) animals in the presence of the cyclooxygenase inhibitor indomethacin (5 μM) and the NO synthase inhibitor N^G-methyl-L-arginine methylester (L-NAME; 300 μM; to reveal endothelium-derived hyperpolarization; **A**) However, the potency of CNP was not altered in vessels from WT versus $gbCNP^{-/-}$ mice (**B**). Vasoconstrictor response to the α_1-adrenoceptor agonist phenylephrine (Phe; **C**) and the thromboxane-A_2 mimetic U46619 (**D**) were not altered by genotype. Data are represented as the mean±SEM. *n*=10. Statistical analysis by two-way analysis of variance across the entire concentration range. Each statistical comparison undertaken has an assigned *P* value (adjusted for multiplicity).

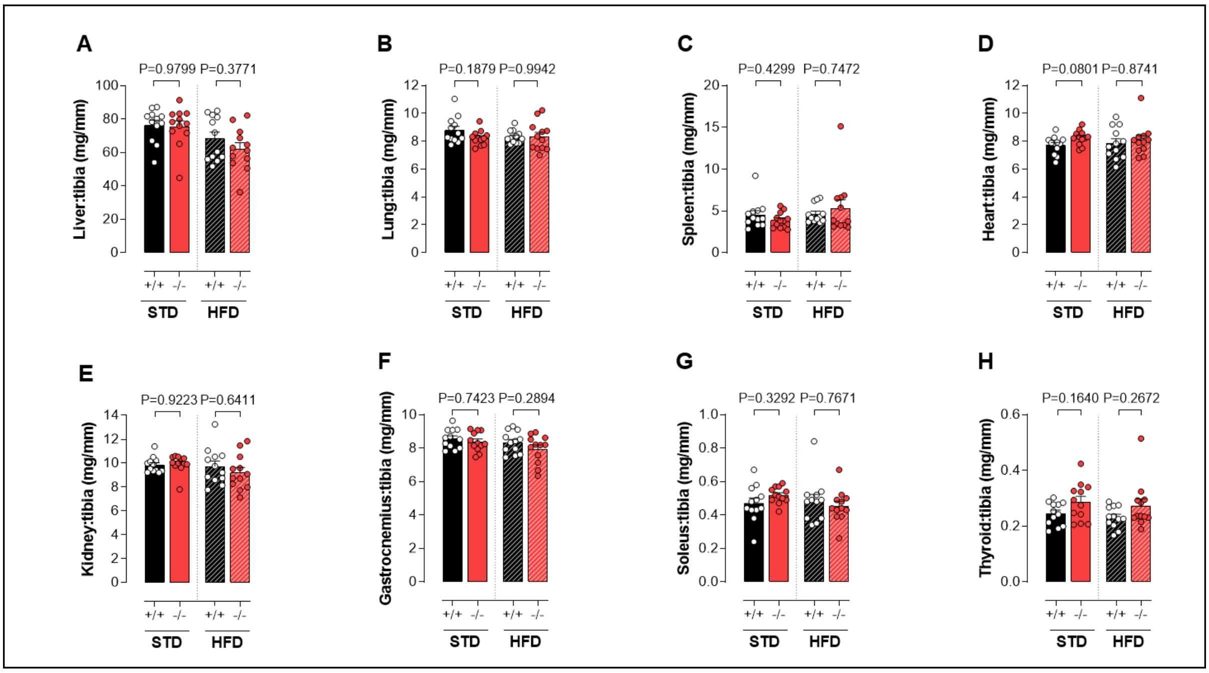

Figure S3: Organ weight is not affected in global CNP$^{-/-}$ mice

Liver (**A**), lung (**B**), spleen (**C**), heart (**D**), kidney (**E**), gastrocnemius (**F**), soleus (**G**), and thyroid (**H**) weight in wild type (WT, +/+) and global CNP KO mice (gbCNP$^{-/-}$, -/-) fed standard chow (STD) or high fat diet (HFD) normalized to tibia length. Data are represented as the mean±SEM. *n*=12. Statistical analysis by two-way analysis of variance with Šídák post hoc test. Each statistical comparison undertaken has an assigned *P* value (adjusted for multiplicity).

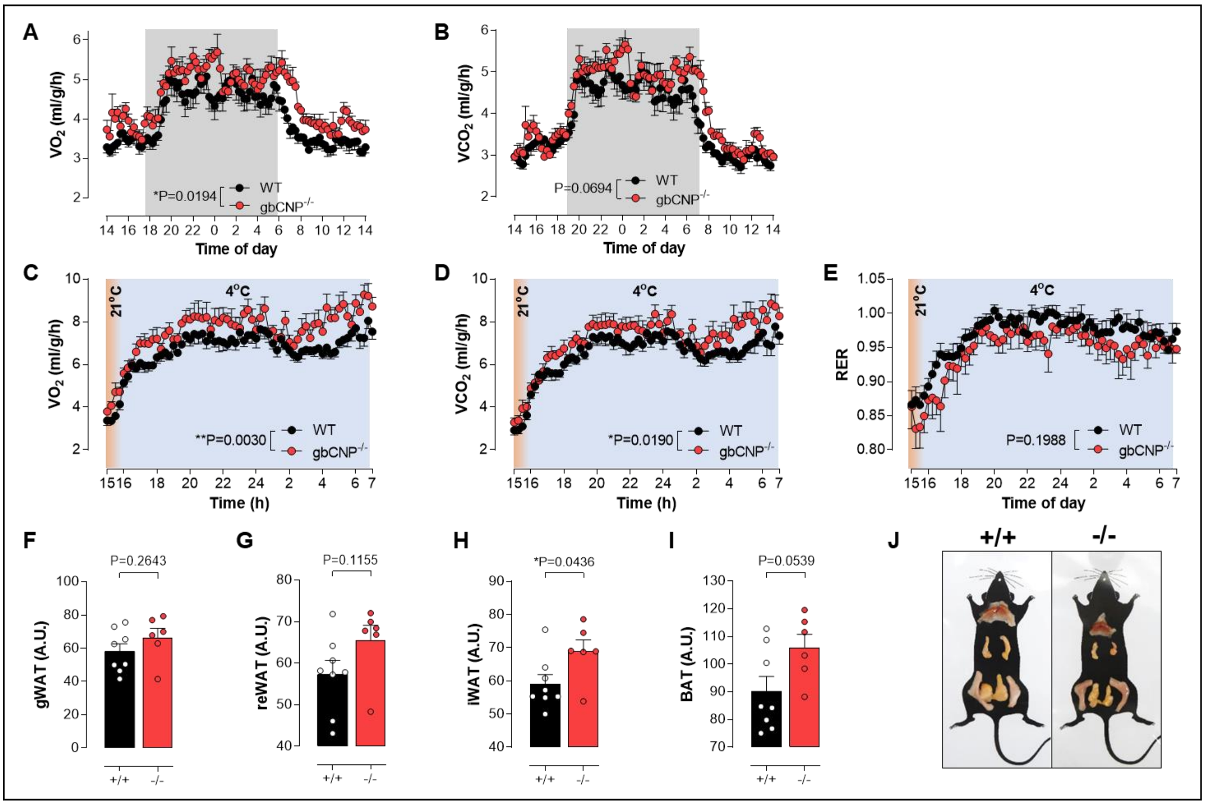

Figure S4: Respiratory exchange rate is altered in in global CNP$^{-/-}$ mice following cold challenge

Oxygen consumption (VO_2; **A**, **C**), carbon dioxide production (VCO_2; **B**, **D**) and respiratory exchange ratio (RER; **E**) in wild type (WT) and global CNP KO (gbCNP$^{-/-}$) mice at 21°C (**A**, **B**) or 4°C (**C**-**E**). Densitometric analyses and representative pictures (**J**) of gonadal (gWAT; **F**), perirenal (reWAT; **G**) and inguinal (iWAT; **H**) white adipose tissue and brown adipose tissue (BAT; **I**) after cold challenge at 4°C. Data are represented as mean±SEM. *n*=6-8. Statistical analysis by two-way repeated measures analysis of variance (**A**-**E**) or two-tailed Student's t-test (**F-I**). Each statistical comparison undertaken has an assigned *P* value (adjusted for multiplicity).

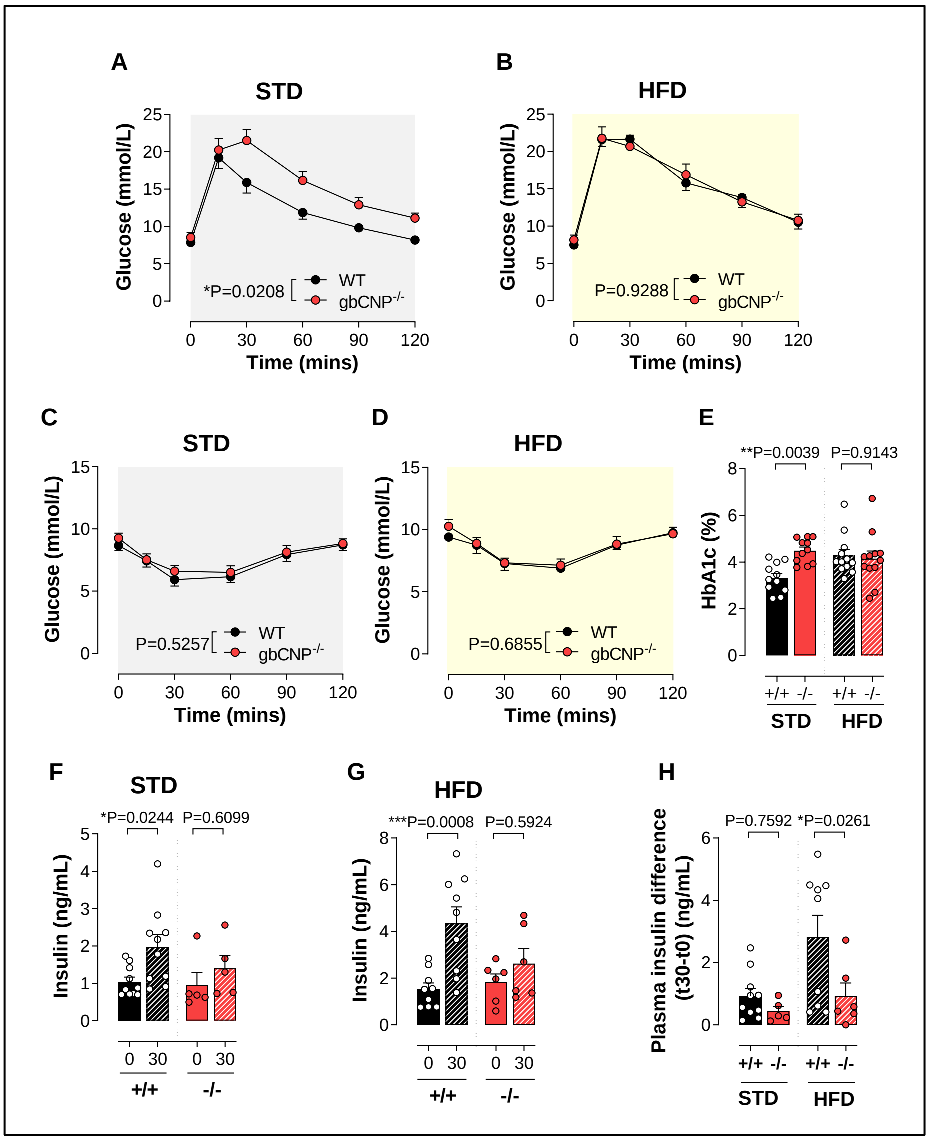

Figure S5: Glucose clearance and insulin release underlie the effects of CNP on lipid handling

Intraperitoneal glucose tolerance test (GTT; **A**, **B**), insulin tolerance test (ITT; **C**, **D**), plasma glycated hemoglobin (HbA1c) levels (**E**), plasma insulin concentration (**F**, **G**), and plasma insulin difference (30 minutes after glucose [2g/kg] challenge; **H**) in wild type (WT, +/+) and global CNP knockout (gbCNP$^{-/-}$, -/-) mice fed standard chow (STD) or high fat diet (HFD). Data are represented as mean±SEM. Statistical analysis by two-way repeated measures analysis of variance (**A**-**D**) or one-way analysis of variance with Šídák *post-hoc* test (**E**-**H**). Each statistical comparison undertaken has an assigned *P* value (adjusted for multiplicity).

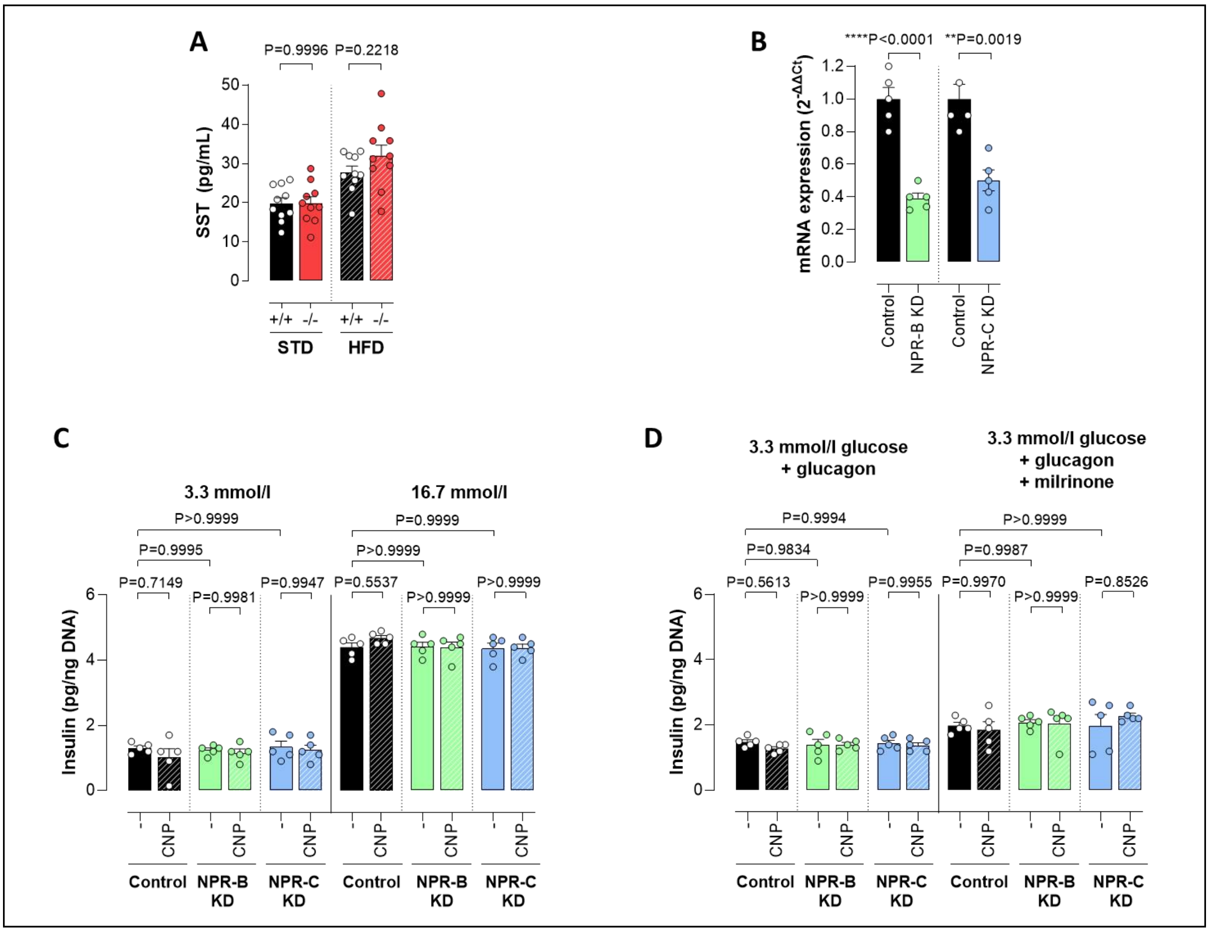

Figure S6: Mechanism of impaired insulin secretion in gbCNP$^{-/-}$

Plasma levels of somatostatin (SST) in wild type (WT, +/+) and global C-type natriuretic peptide (gbCNP$^{-/-}$, -/-) mice on standard chow (STD) or high fat diet (HFD) (**A**). Relative expression of natriuretic peptide receptor (NPR)-B mRNA and NPR-C mRNA in mouse pancreatic islets following siRNA knockdown (KD) compared to administration of a nonsensical RNA sequence (Control) (**B**). Pancreatic islet insulin release in low (3.3 mmol/l) and high (16.7 mmol/l) glucose concentrations under control conditions (-) and after CNP (100 nM) stimulation (**C**). Pancreatic islet insulin release in low (3.3 mmol/l) glucose concentration in the presence of glucagon alone or in combination with the PDE3 inhibitor milrinone in control conditions (-) and after CNP (100 nM) stimulation (**D**). Data are represented as the mean ± SEM. n=5-10. Statistical analysis by two-way analysis of variance with Šídák post hoc test (**A**), two-tailed Student's t-test (**B**) or one-way analysis of variance with Šídák post hoc test (**C** and **D**). Each statistical comparison undertaken has an assigned *P* value (adjusted for multiplicity).

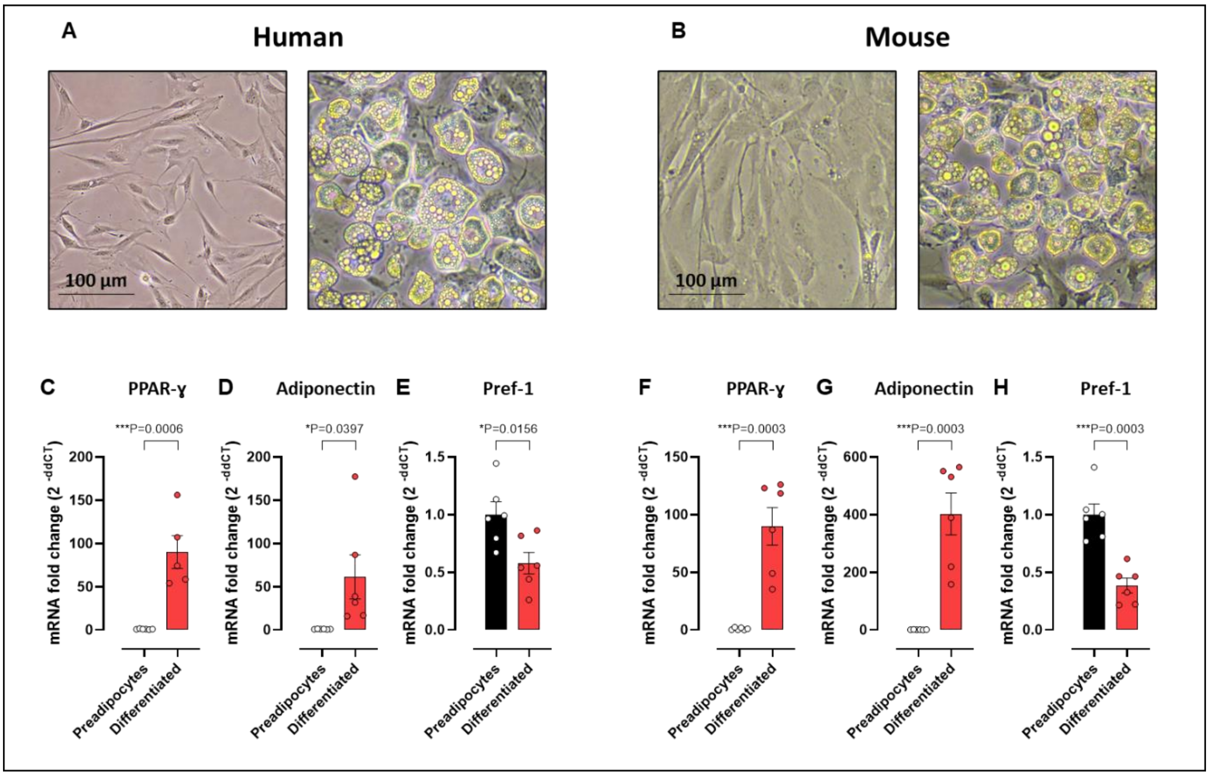

Figure S7: Differentiation status of human and mouse adipocytes *in vitro*

Representative images of preadipocytes (left panel) and differentiated (right panel) human (**A**) and mouse (**B**) adipocytes. mRNA expression of the adipogenic markers peroxisome proliferator-activated receptor (PPAR)-γ (**C, F**), adiponectin (**D, G**) and preadipocyte factor (Pref)-1 (**E, H**) of human (**C**-**E**) and mouse (**F**-**H**) adipocytes. Data are represented as mean±SEM. *n*=6. Statistical analysis by two-tailed Student's t-test. Each statistical comparison undertaken has an assigned *P* value (adjusted for multiplicity).

SUPPLEMENTAL TABLES

Table S1: Heart rate variability and electrocardiogram parameters in wild type (WT) and global CNP knockout ($gbCNP^{-/-}$) mice

	WT	$gbCNP^{-/-}$	P value
**SDNN (ms)**	7.6±0.9	8.3±0.4	0.5030
**HR (bpm)**	531.5±21.0	538.4±15.2	0.7962
**LF (ms^2)**	11.5±3.6	11.2±2.2	0.9467
**HF (ms^2)**	10.0±4.8	10.9±1.7	0.8584
**LF/HF**	1.8±0.2	1.7±0.3	0.8407
**PR interval (ms)**	35.7±0.6	37.9±1.2	0.1226
**P duration (ms)**	15.9±1.5	14.2±1.7	0.4868
**QRS interval (ms)**	9.2±0.1	9.0±0.3	0.5372
**QT interval (ms)**	18.6±0.7	17.4±0.5	0.2035
**QTc (ms)**	54.9±1.7	52.5±1.8	0.3695

Data are represented as the mean ± SEM. *n* = 6. Statistical analysis by two-tailed Student's t-test (adjusted for multiplicity). Abbreviations: bpm, beats per minute; HF, high frequency; HR, heart rate; LF, low frequency; ms, millisecond; SDNN, standard deviation of heart rate interval.

Table S2: Analysis of the relative natriuretic peptide receptor (NPR)-B and NPR-C and C-type natriuretic peptide (CNP) expression in fat depots at 4°C, 21°C and 30°C.

	**4°C**	**21°C**	**30°C**
		**CNP**	
**iWAT**	0.80 ± 0.08	1.00 ± 0.32	0.33 ± 0.11
**reWAT**	n.d.	1.39 ± 0.52	0.77 ± 0.19
**gWAT**	n.d.	1.67 ± 0.78	0.64 ± 0.35
**BAT**	0.56 ± 0.10	0.45 ± 0.10	0.56 ± 0.15
		**NPR-A**	
**iWAT**	1.30 ± 0.20	1.00 ± 0.23	0.61 ± 0.20
**reWAT**	n.d.	1.39 ± 0.23	1.47 ± 0.27
**gWAT**	n.d.	1.50 ± 0.19	1.51 ± 0.08
**BAT**	1.73 ± 0.15	1.25 ± 0.09	1.18 ± 0.28*
		**NPR-B**	
**iWAT**	1.38 ± 0.26	1.00 ± 0.23	1.10 ± 0.18
**reWAT**	n.d.	0.72 ± 0.14	1.16 ± 0.18
**gWAT**	n.d.	0.82 ± 0.08	0.94 ± 0.35
**BAT**	1.27 ± 0.11†,‡	0.83 ± 0.08	0.80 ± 0.03
		**NPR-C**	
**iWAT**	0.57 ± 0.10 †	1.00 ± 0.09	0.85 ± 0.17
**reWAT**	n.d.	0.88 ± 0.16	1.48 ± 0.54
**gWAT**	n.d.	0.96 ± 0.18	1.12 ± 0.24††
**BAT**	0.24 ± 0.05*,‡‡‡	0.51 ± 0.05*	1.34 ± 0.32††

Relative mRNA expression of C-type natriuretic peptide (CNP) and natriuretic peptide receptor (NPR)-A, B and NPR-C in the inguinal (iWAT), peri-renal (reWAT) and gonadal (gWAT) white adipose tissue and brown adipose tissue (BAT) in wild type animals housed at 4°C, 21°C and 30°C normalized to iWAT at 21°C. n.d. = not determined. Data are represented as the mean ± SEM. *n* = 3-6. Statistical analysis by 1-way ANOVA with Šídák post hoc test or two-tailed Student's t-test. *P<0.05, significantly different from iWAT at the same temperature, †P<0.05 and ††P<0.01 significantly different from the same tissue depot at 21°C and ‡P<0.05 and ‡‡‡P<0.001 significantly different from the same tissue depot at 30°C.

Table S3: Heart rate variability and electrocardiogram parameters in wild type (WT) mice before and after infusion of $cANF^{4-23}$

	Control	+ $cANF^{4-23}$	P value
**SDNN (ms)**	8.8±1.0	9.3±0.9	0.7473
**HR (bpm)**	517.5±8.8	517.8±11.3	0.9854
**LF (ms^2)**	13.5±3.9	17.4±4.4	0.5202
**HF (ms^2)**	14.0±6.0	24.8±9.1	0.3439
**LF/HF**	1.5±0.1	1.6±0.2	0.6584
**PR interval (ms)**	36.7±0.8	36.8±1.1	0.9012
**P duration (ms)**	14.4±1.6	14.5±1.7	0.9597
**QRS interval (ms)**	9.7±0.4	9.6±0.5	0.8909
**QT interval (ms)**	17.6±0.6	18.4±1.0	0.5155
**QTc (ms)**	52.2±1.8	54.7±3.5	0.5443

Heart rate variability and electrocardiogram parameters were measured before and during continuous subcutaneous administration of $cANF^{4-23}$ (0.4 mg/kg/d). Data are represented as the mean ± SEM. *n* = 6. Statistical analysis by two-tailed Student's t-test (adjusted for multiplicity). Abbreviations: bpm, beats per minute; HF, high frequency; HR, heart rate; LF, low frequency; ms, millisecond; SDNN, standard deviation of heart rate interval.

Table S4: Patient clinical data

	Lean	Overweight
**Age (y)**	55.5 ± 3.5	54.2 ± 4.0
**Height (m)**	1.7 ± 0.0	1.6 ± 0.0
**Weight (Kg)**	63.9 ± 1.4	72.7 ± 3.3
**BMI (Kg/m^2)**	23.3 ± 0.3	27.5 ± 0.3
**Waist (cm)**	90.4 ± 1.3	91.1 ± 9.9
**Hip (cm)**	101.0 ± 2.2	103.2 ± 11.0
**WHR**	0.9 ± 0.0	0.9 ± 0.1
**% Fat**	27.9 ± 3.2	34.6 ± 4.1
**Fat mass (Kg)**	17.7 ± 2.0	25.1 ± 3.1
**Systolic blood pressure (mmHg)**	120.7 ± 4.7	137.8 ± 5.4
**Diastolic blood pressure (mmHg)**	76.0 ± 2.5	82.8 ± 3.2
**Heart rate (bpm)**	76.8 ± 2.0	74.6 ± 4.8

BMI, body mass index; WHR, waist:hip ratio; blood pressure, BP. Data are represented as the mean ± SEM. *n* = 9-10.

Table S5: Correlation of NT-proCNP with clinical parameters

	Spearman's correlation (r)	95% confidence interval	P value
**Age (y)**	0.2427	-0.2513 to 0.6364	0.3167
**Height (m)**	0.0030	-0.4513 to 0.4561	0.9899
**Weight (Kg)**	0.2002	-0.2789 to 0.5995	0.3975
**BMI (Kg/m^2)**	0.3489	-0.1246 to 0.6929	0.1317
**Waist (cm)**	0.2127	-0.2807 to 0.6172	0.3819
**Hip (cm)**	0.0967	-0.3864 to 0.5381	0.6938
**WHR**	0.2579	-0.2361 to 0.6460	0.2864
**% Fat**	0.4783	0.01627 to 0.7720	*0.0383
**Fat mass (Kg)**	0.5915	0.1737 to 0.8288	**0.0076
**Systolic BP (mmHg)**	0.5356	0.1081 to 0.7959	*0.0149
**Diastolic BP (mmHg)**	0.2319	-0.2480 to 0.6204	0.3253
**Heart rate (bpm)**	-0.1611	-0.5730 to 0.3157	0.4973

BMI, body mass index; WHR, waist:hip ratio; blood pressure, BP. Data are represented as the mean ± SEM. *n* = 19. Statistical analysis by two-tailed Spearman correlation.

Table S6: Analysis of natriuretic peptide receptor (NPR)-B and NPR-C knockdown in human adipocytes.

HUMAN			
	**Control**	**NPR-B KD**	**NPR-C KD**
***NPR-B* mRNA ($2^{-\Delta\Delta Ct}$)**	1.00 ± 0.09	0.34 ± 0.04 (****P<0.001)	0.94 ± 0.05 (P=0.7771)
***NPR-C* mRNA ($2^{-\Delta\Delta Ct}$)**	1.00 ± 0.14	0.97 ± 0.17 (P=0.9795)	0.47 ± 0.07 (*P=0.0234)
**NPR-C protein expression (NPR-C:GAPDH)**	1 ± 0.10	0.86 ± 0.06 (P=0.3112)	0.46 ± 0.03 (***P=0.0001)
**MOUSE**			
***Npr-b* mRNA ($2^{-\Delta\Delta Ct}$)**	1.00 ± 0.26	0.50 ± 0.19 (**P=0.0034)	N/A

Relative expression of natriuretic peptide receptor (NPR)-B mRNA and NPR-C mRNA and protein and in human and mouse adipocytes following siRNA knockdown (KD) compared to administration of a nonsensical RNA sequence (Control). Data are represented as the mean ± SEM. *n* = 6. Statistical analysis by one-way analysis of variance with Šídák post hoc test (human) or two-tailed Student's t-test (mouse). **P<0.01, ***P<0.001 and ****P<0.0001 significantly different from untreated control.

Table S7: Mouse and human primers

Target gene	Primer sequence (5’-3’)	Accession
Mouse Flox Forward	CCTTTATGCCAAGAGAACTTCCAGGAGG	NC_000067.7
Mouse Flox Reverse	TCCTTCCTGACTTCCTTCTGCTCTCTATCC	
Mouse Cre Forward	ATCCGAAAAGAAAACGTTGA	N/A
Mouse Cre Reverse	ATCCAGGTTACGGATATAGT	
Mouse Adiponectin (*Adipoq*) Forward	CAGTGGATCTGACGACACCAA	NM_009605.5
Mouse Adiponectin (*Adipoq*) Reverse	TTAGGACCAAGAAGACCTGCATC	
Mouse CNP (*Nppc*) Forward	CCAACGCGCGCAAATACAAA	NM_010933.5
Mouse CNP (*Nppc*) Reverse	GCACAGAGCAGTTCCCAATC	
Mouse GAPDH (*Gapdh*) Forward	ACCACAGTCCATGCCATCAC	NM_001289726.1<br>NM_008084.3
Mouse GAPDH (*Gapdh*) Reverse	TCCACCACCCTGTTGCTGTA	
Mouse NPR-A (*Npr1*) Forward	TCGATGCTGTACGCTCCTTCC	NM_008727.5
Mouse NPR-A (*Npr1*) Reverse	AACCTTGCCTTTGCCCTTCATTTC	
Mouse NPR-B (*Npr2*) Forward	CCCTGTGCCTTTGACTTGGA	NM_001355466.1
Mouse NPR-B (*Npr2*) Reverse	GCAACAACATTTCCCAGCGA	
Mouse NPR-C (*Npr3*) Forward	CTTGGATGTAGCGCACTATGTC	NM_008728.2<br>NM_001039181.1
Mouse NPR-C (*Npr3*) Reverse	CACAAGGACACGGAATACTC	
Mouse PGC-1α (*Ppargc1a*) Forward	AGCCTGCGAACATATTTGAGAAGA	NM_008904.2
Mouse PGC-1α (*Ppargc1a*) Reverse	TGCTGCATGGTTCTGAGTGC	
Mouse PPAR-γ (*Pparg*) Forward	TTCGCTGATGCACTGCCTAT	NM_011146.3
Mouse PPAR-γ (*Pparg*) Reverse	TGATCGCACTTTGGTATTCTTGG	
Mouse Pref-1 (*Dlk1*) Forward	CTGCAAGGAACCATGGCAGT	NM_001190705.1<br>NM_001190704.1<br>NM_001190703.1<br>NM_010052.5
Mouse Pref-1 (*Dlk1*) Reverse	GCTGGCAGGGAGAACCATTGA	
Mouse Rpl19 (*Rpl19*) Forward	TTGGCGATTTCATTGGTCTCA	NM_009078.2<br>NM_001159483.1
Mouse Rpl19 (*Rpl19*) Reverse	GCTTGCCTCTAGTGTCCTCC	
Mouse UCP-1 (*Ucp-1*) Forward	CAAAGTCCGCCTTCAGATCCAA	NM_009463.3
Mouse UCP-1 (*Ucp-1*) Reverse	AAGCCGGCTGAGATCTTGTT	
Human Adiponectin (*ADIPOQ*) Forward	CTGTTGCTGGGAGCTGTTCT	NM_001177800.2<br>NM_004797.4

Human Adiponectin (*ADIPOQ*) Reverse	GACCAATAAGACCTGGATCTCCT	
Human CNP (*NPPC*) Forward	TACAAAGGAGCCAACAAGAAGG	NM_024409.4
Human CNP (*NPPC*) Reverse	AAAGATGACCTCAGCACAACG	
Human NPR-B (*NPR2*) Forward	ACGGGCGCATTGTGTATATC	NM_003995.3
Human NPR-B (*NPR2*) Reverse	GGGCTCTTATCAGCAGACGA	
Human NPR-C (*NPR3*) Forward	TTGCACACGTCCATCTACAGT	NM_001204375.2<br>NM_000908.4
Human NPR-C (*NPR3*) Reverse	CTCTTCCATGAGCCATCTCCATA	
Human PPAR-γ (*PPARG*) Forward	GGTGACCAGAAGCCTGCATTT	NM_138711.6<br>NM_001330615.4<br>NM_001354667.3<br>NM_001354670.2<br>NM_001374262.3<br>NM_001374266.1
Human PPAR-γ (*PPARG*) Reverse	GCTCCACTTTGATTGCACTTTGGTA	
Human PREF-1 (*DLK1*) Forward	ACAACAGGACCTGCGTGAG	NM_003836.7<br>NM_001317172.2
Human PREF-1 (*DLK1*) Reverse	GAAGTCGCCCCCAATGTCAG	
Human RPL19 (*RPL19*) Forward	TTGGCGATTTCATTGGTCTCA	NM_000981.4<br>NM_001330200.1
Human RPL19 (*RPL19*) Reverse	GCTTGCCTCTAGTGTCCTCC	

REFERENCES

1. Z. Wang *et al.*, The protective effects of the β3 adrenergic receptor agonist BRL37344 against liver steatosis and inflammation in a rat model of high-fat diet-induced nonalcoholic fatty liver disease (NAFLD). *Molecular medicine (Cambridge, Mass.)* **26**, 54 (2020).
2. A. J. Moyes *et al.*, Endothelial C-type natriuretic peptide maintains vascular homeostasis. *J Clin Invest* **124**, 4039-4051 (2014).
3. X. Zhao *et al.*, A comparison of methods for effective differentiation of the frozen-thawed 3T3-L1 cells. *Anal Biochem* **568**, 57-64 (2019).
4. Y. X. Zhu *et al.*, Protocol for in vivo and ex vivo assessments of glucose-stimulated insulin secretion in mouse islet β cells. *STAR protocols* **2**, 100728 (2021).
